# Revealing the pharmacological mechanisms of nao-an dropping pill in preventing and treating ischemic stroke via the PI3K/Akt/eNOS and Nrf2/HO-1 pathways

**DOI:** 10.1038/s41598-024-61770-4

**Published:** 2024-05-16

**Authors:** Chen Wang, Zhe-Ming Xiong, You-Quan Cong, Zi-Yao Li, Yi Xie, Ying-Xiao Wang, Hui-Min Zhou, Yan-Fang Yang, Jing-Jing Liu, He-Zhen Wu

**Affiliations:** 1 College of Pharmacy, Hubei University of Chinese Medicine, Wuhan, 430065 China; 2 Leiyunshang Pharmaceutical Group Co., Ltd, Suzhou, 215009 China; 3 Key Laboratory of Traditional Chinese Medicine Resources and Chemistry of Hubei Province, Wuhan, 430065 China; 4 Modern Engineering Research Center of Traditional Chinese Medicine and Ethnic Medicine of Hubei Province, Wuhan, 430065 China

**Keywords:** Nao-an dropping pill, Ischemic stroke, Network pharmacology, OGD/R, MCAO/R, Mechanism, Drug discovery, Molecular biology

## Abstract

Nao-an Dropping Pill (NADP) is a Chinese patent medicine which commonly used in clinic for ischemic stroke (IS). However, the material basis and mechanism of its prevention or treatment of IS are unclear, then we carried out this study. 52 incoming blood components were resolved by UHPLC-MS/MS from rat serum, including 45 prototype components. The potential active prototype components hydroxysafflor yellow A, ginsenoside F1, quercetin, ferulic acid and caffeic acid screened by network pharmacology showed strongly binding ability with PIK3CA, AKT1, NOS3, NFE2L2 and HMOX1 by molecular docking. In vitro oxygen–glucose deprivation/reperfusion (OGD/R) experimental results showed that NADP protected HA1800 cells from OGD/R-induced apoptosis by affecting the release of LDH, production of NO, and content of SOD and MDA. Meanwhile, NADP could improve behavioral of middle cerebral artery occlusion/reperfusion (MCAO/R) rats, reduce ischemic area of cerebral cortex, decrease brain water and glutamate (Glu) content, and improve oxidative stress response. Immunohistochemical results showed that NADP significantly regulated the expression of PI3K, Akt, p-Akt, eNOS, p-eNOS, Nrf2 and HO-1 in cerebral ischemic tissues. The results suggested that NADP protects brain tissues and ameliorates oxidative stress damage to brain tissues from IS by regulating PI3K/Akt/eNOS and Nrf2/HO-1 signaling pathways.

## Introduction

Stroke, a major chronic non-communicable disease that seriously jeopardizes the health of the nation due to the interruption of cerebral blood flow caused by embolism (IS) or rupture (hemorrhagic stroke) of blood vessels in the brain, resulting in damage to brain tissues, physical disability, and multifunctional impairment^1^. The incidence of IS accounts for about 70% of the total incidence of stroke, with the characteristics of high morbidity, high mortality, high disability rate, high recurrence rate and high economic burden^2,3^. The pathogens of IS are complex, with energy metabolism disorder, amino acid toxicity, free radical damage, inflammation, penumbra depolarization and apoptosis collectively participating in the pathogenesis of IS^4,5^. Various etiological factors interact with each other, leading to the occurrence or recurrence of the disease. Starting from the above factors, the goal of preventing or treating IS can be achieved.

At present, for the prevention and treatment of IS, Chinese and Western medicine have their own advantages. Western medicine usually takes the compound monomer as the effective ingredient, and the action target is specialized, while traditional Chinese medicine is generally composed of single or multiple herbs, with multiple targets to play a role, characterized by versatility and integrity^6–9^. Since IS is a disease caused by a variety of etiological factors, traditional Chinese medicine and its compound prescription can be used in different routes of administration according to the characteristics of the disease, and flexibly increase or decrease the drug dose according to the symptoms, which provides distinctive and unique advantages compared with Western medicine treatment^10,11^. NADP is composed of *Ligusticum chuanxiong* Hort., *Angelica sinensis* (Oliv.) Diels, *Carthamus tinctorius* L., *Panax ginseng* C. A. Meyer and Borneol. Its prescription was improved from the representative compound formula "Buyang Huanwu Decoction" for the prevention and treatment of IS in the work of Wang Qingren, a famous doctor in the Qing Dynasty of China, entitled "Corrections on the Errors of Medical Works"^12^. NADP has the effects of promoting blood circulation and removing blood stasis, activating qi and collateral, clinically used for the secondary prevention of IS and migraine with definite curative effect, and has been used as medicine for several decades^13,14^. However, due to the complexity of traditional Chinese medicine and its compound prescription, the material basis and mechanism of NADP in preventing and treating of IS have not been reported.

Based on serum pharmacochemistry, this study investigated the efficacy of NADP in preventing and treating IS by means of network pharmacology, in vitro cellular experiments and in vivo animal experiments, and explored the material basis and mechanism NADP in preventing and treating IS, so as to provide scientific basis for the clinical use of NADP.

## Materials and methods

### Chemical and reagents

NADP extract powder was provided by Leiyunshang Pharmaceutical Group Co., Ltd. (Suzhou, China). Compound Danshen Dropping Pill (CDDP, Lot. 201227) was purchased from Tasly Pharmaceutical Group Co., Ltd. (Tianjing, China). Ginkgo Leaf Tablet (GLT, Lot. 20121891) was purchased from Yangtze River Pharmaceutical Group (Taizhou, China). Pentobarbital sodium (Lot. 86-01-22) was purchased from Shanghai Chemical Reagent Purchasing and Supply Station (Shanghai, China). 2,3,5-Triphenyltetrazolium chloride (TTC, Lot. S19026) was purchased from yuanye Bio-Technology Co., Ltd. (Shanghai, China). PI3 Kinase p110 Beta antibody (Lot. 20584-1-AP), NFE2L2 Polyclonal antibody (Lot. 16396-1-AP) and HMOX1 Polyclonal antibody (Lot. 10701-1-AP) were purchased from Proteintech Group, Inc (Wuhan, China). Akt Antibody (Lot. AY0420) was purchased from Abways technology (Shanghai, China), Phospho-AKT1-S473 antibody (Lot. AP0140) and Phospho-eNOS-S1177 antibody (Lot. AP0421) were purchased from Abclonal technology (Wuhan, China).eNOS Polyclonal Antibody (Lot. PAB48019) was purchased from Bioswamp (Wuhan, China). BCA protein colorimetric assay kit (E-BC-K318-M), lactate dehydrogenase (LDH) activity assay kit (E-BC-K046-M), nitric oxide (NO) colorimetric assay kit (E-BC-K135-M), glutamate (Glu) colorimetric assay kit (E-BC-K118-S), malondialdehyde (MDA) colorimetric assay kit (In vitro: E-BC-K028-M; In vivo: E-BC-K025-M), superoxide dismutase (SOD) activity assay kit (E-BC-022-M), myeloperoxidase (MPO) activity assay kit (E-BC-K074-M) and H&E staining kit (E-IR-R117) were purchased from Elabscience Biotechnology Co., Ltd. (Wuhan, China).

### Identification of incoming blood components of NADP

Female SD rats (200 ± 20 g, the pre-experimental results showed that more blood components were detected in the drug-containing serum of female rats compared to male) were obtained from the Hubei Provincial Center for Disease Control and Prevention (License number: SYXK (e) 2017–0067). Rats were fed in SPF barrier system with constant temperature and humidity at 22℃, fed and drank freely for seven days. There were 3 rats in each of the administration and blank groups. After fasting for 18 h, the rats in both the administration group and blank group were gavaged at a dose of 1 mL/100 g. The administration group was given NADP (1.5 g/mL), and the blank group was given saline (0.9%). Blood was collected from the orbital venous plexus at 5 min and 30 min after gavage and stand at 4℃ for 2 h. The collected blood was centrifuged at 4,000 rpm, 4℃ for 10 min, and the upper serum of the two time points was taken and combined as blank serum and NADP-containing serum.

800.0 μL of NADP-containing serum was fully mixed with 700.0 μL anhydrous ethanol, the mixture was centrifuged at 10,000 rpm at 4℃ for 10 min. The protein-free supernatant was dried on nitrogen blower. The residue I was ultrasonically dissolved in 150.0 μL anhydrous ethanol, filtered through a 0.22 μm microporous membrane, then dried on nitrogen blower, and the residue II was ultrasonically dissolved in 150.0 μL methanol. 10.0 μL of drug-containing serum sample was taken for UHPLC-MS/MS analysis according to the gradient elution procedure in Table 1. The blank serum was processed and analyzed in the same way.

**Table 1:** The gradient elution program of UHPLC-MS/MS.

Time (min)	Mobile phase A (%)	Mobile phase B (%)
0–7	95 → 90	5 → 10
7–25	90 → 82	10 → 18
25–30	82 → 70	18 → 30
30–50	70 → 50	30 → 50
50–53	50 → 44	50 → 56
53–63	44 → 39	56 → 61
63–73	39 → 5	61 → 95
73–75	5	95

The primary mass spectrometry information and secondary mass spectrometry fragment data of the differential peaks were compared with the mass spectrometry information of the standards and standard fragment ion in vitro to identify the structure of the incoming blood prototype components^15^.

### Network pharmacology analysis based on incoming blood components

#### Databases and software

PubChem database (https://pubchem.ncbi.nlm.nih.gov/), SwissTargetPrediction (http://www.swisstargetprediction.ch/), PharmMapper (http://www.lilab-ecust.cn/pharmmapper/), GeneCards database (https://www.genecards.org/), OmicShare (https://www.omicshare.com/), STRING (https://www.string-db.org/), PDB database (https://www.rcsb.org/), Cytoscape 3.9.1 software, Discovery Studio 2019 Client software.

#### Prediction of intersection targets

All the incoming blood prototype components were searched in the PubChem database, and the SDF files of their chemical structures were downloaded and imported into SwissTargetPrediction and PharmMapper to match with the internal pharmacophore modeling database to predict the potential targets of each component. All the targets of IS were searched with "Ischemic Stroke" as keyword in GeneCards database, and relevance score ≥ 15.00 was selected as potential target of IS^16^. The intersection targets of the two target sets could be considered as the potential targets of NADP in preventing and treating IS.

#### Enrichment analysis of GO/KEGG

The intersected targets were imported into the OmicShare analysis platform, and the "GO Enrichment Analysis" and "KEGG Enrichment Analysis" items were selected to analyze the gene ontology (GO) function and kyoto encyclopedia of genes and genomes (KEGG) signaling pathway enrichment of the potential targets, and "Ensemble_104 or 51" and "Homo sapiens" were used as the version and species, the results were sorted in ascending order according to *P* value, and the top 20 signaling pathways were filtered as key pathways for analysis^17^.

#### Construction of PPI and C-T-P network

Protein-Protein Interaction (PPI) and its derived networks are important in most biological functions and processes, and most proteins seem to activate their functions through interactions. The analysis of network is helpful to systematically study the molecular mechanism of disease and discover new drug targets^18^. The PPI network was constructed by importing the intersection targets into the STRING to screen the key target genes with the highest Degree value, the closest interactions and high confidence.

The incoming blood prototype components, intersection targets and top 20 signaling pathways were imported into Cytoscape 3.9.1 software to construct a Component-Target-Pathway (C-T-P) network, and the correlation between components and targets was judged by the Degree of association between the nodes in the network^19^.

#### Validation of high-throughput molecular docking

The core targets screened above were searched in PDB database, then the PDB files of the protein crystal 3D structures most related to the disease were obtained^20^. The PDB files and SDF files were imported into Discovery Studio 2019 Client molecular modeling software for virtual molecular docking to verify the binding activity of components to proteins^21^.

The protein structure was imported into Discovery Studio 2019 Client software, and the non-standard residue (Hetatm) and ligand groups were removed, then the protein structure was cleaned. "From Receptor Cavities" was clicked to bring up all the receptor sites, and "From Current Selection" was clicked to select a single site and adjust the radius to 10.00. "Docking Preferences" was adjusted to "User Specified", and "Max Hits to Save" was set as 10.00, then dock with protein structure as receptor and component structure as ligand.

### Effect of NADP on HA1800 cells with OGD/R-induced damage

#### Cytotoxicity of NADP on HA1800 cells

The density of normal human brain astrocyte cells (HA1800, obtained from ATCC, verified by STR) cell in logarithmic growth phase was adjusted to 1 × 10^5^ cells/mL, the cells were inoculated into 96-well plates according to 100.0 μL/well. The blank group (without HA1800 cells), control group and administration group (containing 5%, 10%, 15%, 20%, 25%, 30%, 40%, 50% NADP-containing serum) were set up, with 6 replicate wells in each group. After HA1800 cells were attached to the wall, the blank and control groups were replaced with fresh 200.0 μL of DMEM culture medium (Lot. GIBCO C11965500BT), and the administration group was replaced with 200.0 μL of culture medium with different ratios of NADP-containing serum (NADP-containing serum was prepared with DMEM culture medium). After 24 h of co-incubation of NADP with HA1800 cells, the cell activity was detected by *MTT* assay, and the optical density (OD) of each well was detected at the wavelength of 490 nm on microplate reader. Cell activity (%) = (OD_administration_ - ODblank)/(OD_control_ - OD_blank_) × 100%. The experiment was repeated 3 times.

#### HA1800 cells hypoxia and glucose deficiency injury induced by OGD/R

After the density of HA1800 cells in logarithmic growth phase was adjusted to 1 × 10^5^ cells/mL, the cells were inoculated into 96-well plates according to 100.0 μL/well. The control group, model group (OGD/R) and administration group (OGD/R & NADP-containing serum) were set up, with 6 replicate wells in each group. After cells were attached to the wall, the control and model groups were replaced with 200.0 μL of DMEM culture medium (containing 5% blank serum), and the administration group was replaced with 200.0 μL of culture medium with low (5%), medium (10%) and high (15%) of NADP-containing serum.

After 24 h of co-incubation of NADP with HA1800 cells, the control group was replaced with 200.0 μL fresh DMEM culture medium, the model and administration groups were replaced with 200.0 μL DMEM without glucose (Lot. MA0582, purchased from MeilunBio®, Dalian, China). Then the model and administration groups were placed in an anaerobic system containing 95% N_2_ and 5% CO_2_, and cultured at 37℃ for 4 h of oxygen–glucose deprivation. After 4 h of oxygen and glucose deprivation, the control and model groups were replaced with 200.0 μL of DMEM culture medium (containing 5% blank serum), and the administration group was replaced with 200.0 μL of culture medium with low (5%), medium (10%) and high (15%) of NADP-containing serum. Each group was cultured at 4℃ for 24 h to form oxygen-glucose reperfusion injury^22^.

#### Evaluation of cell morphology and survival rate

Evaluation of morphological changes of HA1800 cells in each group after 24 h of oxygen-glucose reperfusion. *MTT* assay was used to detect the H1800 cell survival rate. Cell activity (%) = (OD_model_/_administration_ - OD_blank_)/(OD_control_ - OD_blank_) × 100%. The experiment was repeated 3 times.

#### Detection of LDH, NO and oxidative stress factors

Extraction of culture supernatants and cell proteins of HA1800 cells in each group after 24 h of oxygen-glucose reperfusion. BCA kit was used to detect the concentration of each sample, then the release of LDH, content of NO, MDA and SOD were determined according to the instructions of the kits. The detection was repeated 3 times.

### Effect of NADP on SD rats with MCAO/R-induced damage

#### MCAO/R-induced cerebral ischemia–reperfusion injury in SD rats

Male SD rats (270–280 g) were obtained from the Hubei Provincial Center for Disease Control and Prevention (License number: SYXK (e) 2017–0067). Rats were fed in SPF barrier system with constant temperature at 22℃ and relative humidity at 50% to 60%, fed and drank freely for seven days. This research was approved by the Animal center of Hubei University of Chinese Medicine (approval number: HUCMS202210001). This in vivo experiment was reported in accordance with the ARRIVE guidelines. The research was carried out according to the recommendations of Animal Care and Use Committee of Institute of Materia Medica, China.

**I**. Under aseptic conditions, 2% pentobarbital sodium solution was injected into the abdominal cavity of rats at the dose of 0.2 mL/100 g. After the rats were thoroughly anesthetized, the neck skin was shaved and clipped, and the muscle tissue was bluntly stripped to expose the common carotid artery (CCA), internal carotid artery (ICA) and external carotid artery (ECA). The skin was sutured and sterilized with 75% ethanol, and the rats were resuscitated at a constant temperature of 37℃.

**II**. After exposing the CCA, ICA and ECA in rats, cerebral ischemia was induced by blocking the blood flow of middle cerebral artery by the MCAO/R approach. Restoration of blood flow to cause reperfusion injury after 40 min of cerebral ischemia. The skin was sutured and sterilized with 75% ethanol, and the rats were resuscitated at a constant temperature of 37℃^23^.

The sham group (step **I** only) and model group (step I & II) were set, with 10 rats in each group. Both the sham and model groups drank 0.9% saline freely.

*Preventive administration*. The NADP-Low (30.0 mg/kg), NADP-Medium (60.0 mg/kg), NADP-High (120.0 mg/kg), positive administration CDDP (72.9 mg/kg) and GLT (108.0 mg/kg) were set, with 10 rats in each group. All the preventive administration groups performed step I & II and administered once daily for 7 consecutive days, with the last administered within 30 min before MCAO/R.

*Therapeutic administration*. The NADP-Low (30.0 mg/kg), NADP-Medium (60.0 mg/kg), NADP-High (120.0 mg/kg), positive administration CDDP (72.9 mg/kg) and GLT (108.0 mg/kg) were set, with 10 rats in each group. All the therapeutic administration groups performed step I & II and administered once after the MCAO/R rats were completely awakened from anesthesia.

### Evaluation of Behavioral, brain water content and ischemic area in rats

Behavioral score of rats after 24 h of reperfusion was performed using the Zea Longa scoring mean^24^. 0 pts: No neurological deficits; 1 pts: Inability to fully extend the front claw on the paralyzed side; 2 pts: Circling to the paralyzed side while walking; 3 pts: Tipping and rolling to the paralyzed side while walking; 4 pts: Inability to walk independently, loss of consciousness. All the rats were recorded behavior.

Rats were anesthetized after 24 h of reperfusion, then blood was taken from the abdominal aorta to death. The brains of rats in each group were carefully peeled off, rinsed with 0.9% saline until the surface was free of blood stains, and weighed and counted as wet weight (W_w_) after the water was absorbed by filter paper. Brain tissues were dried at 95℃ to constant weight, which was counted as dry weight (W_d_)^25^. Brain water content = (W_w_ - W_d_)/W_w_ × 100%. 3 brains were taken for drying.

The cleaned brain tissues were frozen at − 20℃ for 20 min until the brains were stereotyped, then cut into 5 slices about 2 mm thickness and placed in a 6-well plate with 2.0 mL of 2% TTC staining solution in each well. After the brain slices were stained without light for 30 min, the surface dye was dried and photographed under the blue background, and the ischemic area of brain slices was analyzed by Image-Pro Plus 6.0 software. 4 brains were taken for TTC staining.

### H&E staining for pathologic damage in ischemic area of cerebral cortex

The cleaned brain tissues were fixed in 4% paraformaldehyde for more than 24 h, then removed for paraffin embedding. Cerebral cortex on the ischemic side of the brains were processed by H&E kit, and images were acquired and analyzed.

### Detection of excitatory amino acid and oxidative stress factors

The cerebral cortex tissues on the ischemic side of the brains were fully homogenized in ice water bath at the ratio of brain tissue (g): saline (mL) = 1: 9^26^. The homogenate was centrifuged at 10,000 ×g at 4℃ for 10 min. BCA kit was used to detect the concentration of supernatant, then the content of Glu, MDA, MPO and SOD were determined according to the instructions of the kits. The detection was repeated 3 times.

### Immunohistochemical detection of PI3K/Akt/eNOS and Nrf2/HO-1 expression

Paraffin-embedded cerebral cortex tissues on the ischemic side of the brains were taken and the expressions of PI3K, Akt, p-Akt, eNOS, p-eNOS, Nrf2 and HO-1 were measured by immunohistochemistry. The experiments were repeated 3 times.

#### Statistical analysis

The data obtained in the study were analyzed by GraphPad Prism 8 and Image J software, expressed as mean ± SD, and statistically analyzed by one-way ANOVA, which was considered statistically significant when *P* < 0.05.

## Results

### Analysis of serum components

By comparing the primary mass spectra of blank serum and NADP-containing serum, a total of 49 differential peaks were screened out, in which the differential peaks in negative ion mode were consistent with that in positive ion mode, and the positive ion mode included more differential peaks, so only the differential peaks of positive ion mode were analyzed. The total ion flow diagram of positive ion mode was shown in Fig. 1. 45 incoming blood prototype components were identified, and 7 components were likely to be in vivo metabolites, as shown in Table 2. The cleavage modes of the incoming blood prototype components were shown in supplementary Figs. 1–28.

**Figure 1:**
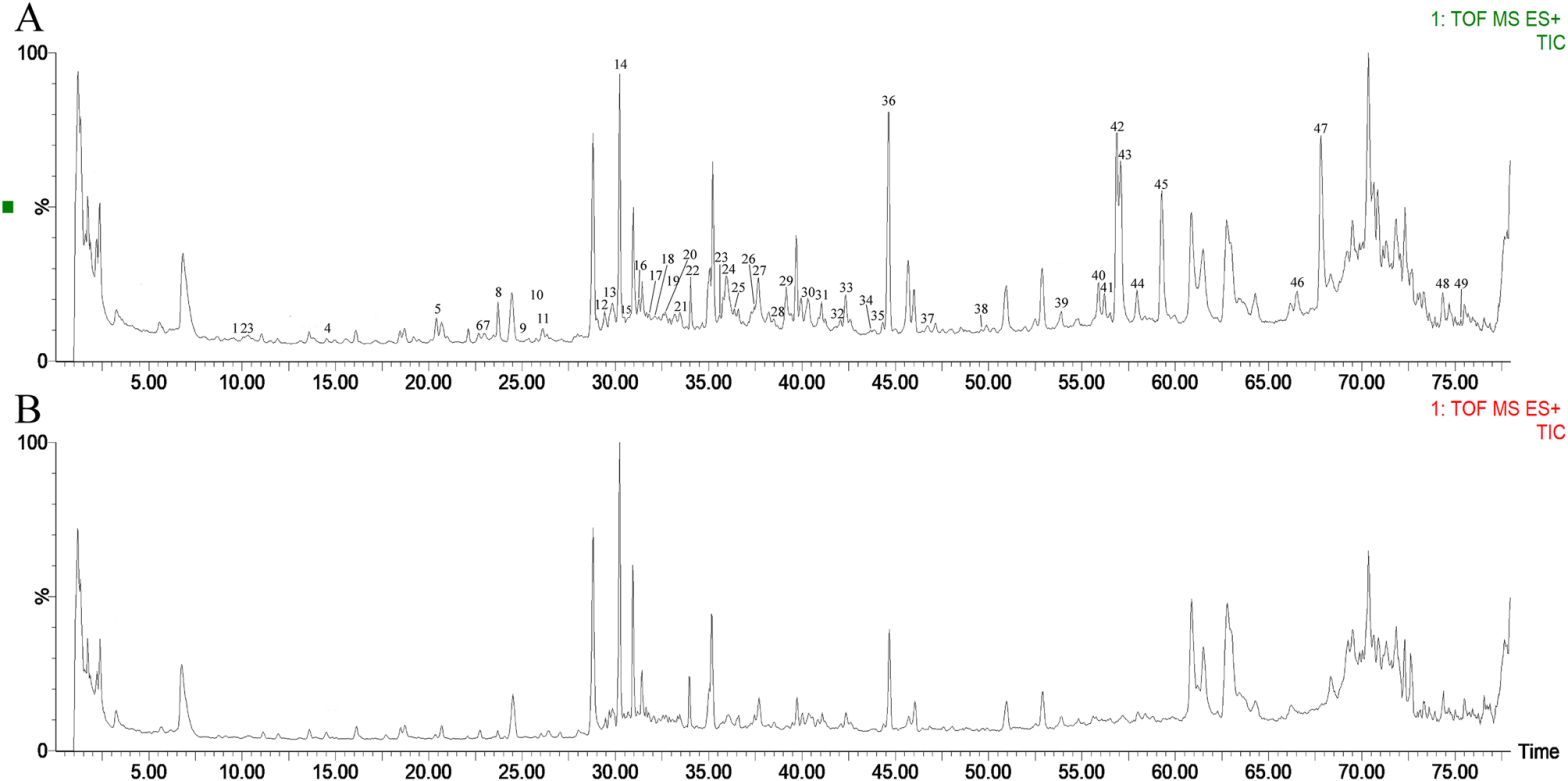
Comparison of UHPLC-MS/MS chromatograms of drug-containing serum and blank serum in positive ion mode (n = 3). (**A**) Drug-containing serum. (**B**) Blank serum.

**Table 2:** The identification information of differential peaks in positive ion mode.

No	Rt (min)	MW	Incoming blood fragment ion (*m/z*)	Standard fragment ion in vitro (*m/z*)	MF	Component identification	Identification basis
1	9.83	516	517.4569, 500.2416, 320.2623, 164.0401	517, 500, 320, 164, 145, 117	C_25_H_24_O_12_	(-)-3,5-Dicaffeoylquinic acid	PubChem
2	10.053	218	219.2474, 189.1329, 175.0368, 151.0363, 123.0438	189, 175, 151, 123, 95, 67	C_13_H_14_O_3_	Chuanxiongol	PubChem
3	10.329	224	225.2101, 208.0619, 207.0536, 175.0341	225.2180, 208.0682, 207.0645, 175.0385	C_11_H_12_O_5_	Sinapic acid	PubChem
4	14.518	947	948.1574, 946.5432, 502.2791	1059.5316, 1005.5594, 946.5474, 945.5426, 783.4, 637.4, 502.2706	C_48_H_82_O_18_	Ginsenoside Re	Standard
5	20.415	354	355.3087, 192.0579, 173.1	355.3099, 192.0589, 191.0555, 173.0446, 161.0271	C_16_H_18_O_9_	Chlorogenic acid	Standard
6	22.655	639	683.4335, 640.8834, 637.4305, 161.0462	683.4364, 640.8885, 637.4310, 161.0461	C_36_H_63_O_9_	Ginsenoside Rh1	PubChem
7	23.019	194	217.1074, 195.2689, 177.0541, 149.0611	444.1522, 442.1467, 313.1045, 296.0966, 295.0937	C_12_H_18_O_2_	Cnidilide	PubChem
8	23.707	801	859.5078, 801.4899, 799.4798	914.4766, 913.4766, 860.5069, 859.5063, 799.4809	C_42_H_72_O_14_	Ginsenoside Rg1	Standard
9	25.346	252	253.2196, 175.1207, 137.1331, 119.0881, 105.4900	175, 147, 137, 119, 105, 91	C_12_H_12_O_6_	Senkyunolide A	PubChem
10	25.758	278	287.0341, 279.4276, 258.0552, 174.9583	287.0549, 289.0609, 286.0467, 258.0515, 165.0191, 153.0209	C_18_H_30_O_2_	Linoleic Acid	PubChem
11	26.122	318	332.1576, 319.2329, 314.1713	332, 314, 303, 289, 273, 261, 245	C_15_H_10_O_8_	Quercetagetin	PubChem
12	29.414	180	181.1536, 179.0356, 163.0327, 145.0269	181.1491, 179.0337, 163.0386, 145.0258	C_9_H_8_O_4_	Caffeic acid	Standard
13	29.846	270	271.2394, 158.0098, 153.0226, 118.0353	271.2316, 158.0122, 153.0194, 118.0369, 117.0329, 107.0169	C_15_H_10_O_5_	Apigenin	PubChem
14	30.224	441	442.3974, 313.1067, 295.1033	444.1522, 442.3914, 313.1045, 296.0966, 295.0937	C_19_H_19_N_7_O_6_	Folic acid	Standard
15	30.689	204	205.2285, 187.0682, 168.0583, 144.0552	205.2259, 187.0745, 168.0574, 144.0573, 131.0493, 115.0541	C_12_H_12_O_3_	Senkyunolide B	PubChem
16	31.276	1079	1080.2693,1077.2191,945.6562, 915.0120, 783.3876	1077, 945, 915, 784, 783, 621, 459	C_53_H_90_O_22_	Ginsenoside Rb2	PubChem
17	31.809	194	195.1811,178.0204, 149.0534, 134.0372, 93.0456,	193.0500, 178.0263, 149.0597, 134.0361	C_10_H_10_O_4_	Ferulic acid	Standard
17	31.809	222	223.2372, 177.0876, 167.0346, 149.0262	223.2365, 177.0899, 167,0387, 149.0227, 121.0278	C_12_H_14_O_4_	Senkyunolide D	PubChem
17	31.809	957	958.1061, 439.3588, 204.1950, 146.0611	440.3758, 439.3561, 204.1911, 146.0502	C_48_H_76_O_19_	Ginsenoside Ro	PubChem
18	32.12	152	153.1488, 136.0111, 123.0416, 108.0208	153.1401, 136.0166, 123.0453, 108.0218, 107.0504	C_8_H_8_O_3_	Vanillin	PubChem
19	32.551	801	801.0148, 653.4359, 635.3390, 421.3433	801, 653, 635, 491, 421.3476	C_42_H_72_O_14_	Pseudoginsenoside FII	PubChem
20	32.673	766	811.4855, 767.9964, 113.0220	811.4849, 113.0236, 101.0235, 71.0128	C_42_H_70_O_12_	Ginsenoside Rk1	PubChem
21	33.448	188	189.2206, 191.0796, 171.0637	191, 189, 171	C_12_H_12_O_2_	Butylidenephthalide	PubChem
22	34.035	801	802.0185, 637.6663, 475.1632	637.66, 475.100, 391.3	C_42_H_72_O_14_	Ginsenoside Rf	Standard
23	35.587	612	613.5392, 611.2476, 491.2407, 473.2178	611, 491, 473	C_27_H_32_O_16_	Hydroxysafflor yellow A	Standard
24	35.931	206	207.1037, 189.0935, 171.0048, 161.0967	207.1031, 189, 171, 161	C_12_H_14_O_3_	Senkyunolide F	PubChem
25	36.363	536	536.4357, 445.3426, 444.3860, 399.3277, 281.3251	537.4367, 445.3420, 444.3885, 399.3213, 281.3202, 255.1976	C_40_H_56_	Beta-carotene	PubChem
26	37.449	446	447.0973, 287.0644, 269.0659	447.0995, 287.0681, 269.0646	C_21_H_18_O_11_	Baicalin	Standard
27	37.672	785	786.016, 784.4360, 639.0477, 621.3923	786, 784, 639, 621, 477	C_42_H_72_O_13_	Ginsenoside Rg2	PubChem
28	38.481	312	313.0179, 311.1792, 248.0103, 188.0671	313.0100, 311.1759, 248.0175, 212.0596, 188.0608, 184.0280	C_20_H_40_O_2_	Arachic acid	PubChem
29	39.156	302	303.0567, 273.0478, 257.0432	303.0599, 301.0347, 273.0417, 257.0447, 229.0494	C_15_H_10_O_7_	Quercetin	Standard
30	40.309	946	946.5626, 945.5430, 783.5047	946.5453, 945.5447, 783.4924, 621.4390	C_48_H_82_O_19_	Ginsenoside Rd	PubChem
31	41.051	353	354.9872, 275.1769, 189.6710	354.999, 275.171, 189.679, 188.578, 149.288	C_20_H_19_NO_5_	Fumarine	PubChem
32	42.05	1109	1109.6087,767.4935, 667.2260	1109.6099,767.4936,667.2288,649.2183,487.1653, 425.3771, 325.1124	C_54_H_92_O_23_	Ginsenoside Rb1	Standard
33	42.326	270	271.2071, 270.2307, 267.0383, 239.0308	267.0305, 239.0351, 223.0479, 171.0668, 139.0234, 137.0073	C_15_H_10_O_5_	Baicalein	PubChem
34	43.689	380	413.2333, 381.2488, 335.0841, 191.1249	413.2, 381.2, 335, 191.1, 149	C_24_H_28_O_4_	Levistolid A	PubChem
35	44.31	372	432.1530, 431.1595, 373.3607, 371.1385, 193.0802	432.1578, 431.1552, 371.1335, 209.0810, 193.0865, 161.0597	C_17_H_24_O_9_	Syringin	PubChem
36	44.654	–	–	–	–	metabolite	
37	46.759	414	415.7030, 156.9436	156.9415, 95.0796, 93.0495	C_29_H_50_O	Beta-sitosterol	PubChem
37	46.759	514	515.2279, 514.2579, 469.2210, 385.1409	515.2263, 469.2283, 385.1443, 355.2161, 343.1087, 323.1160	C_28_H_34_O_9_	Schisantherin B	PubChem
38	49.587	412	413.0446, 159.0386	413.0422, 159.0344, 83.0100, 69.0501, 57.0491	C_29_H_48_O	Stigmasterol	Standard
39	53.9	263	264.1465, 234.0916, 194.0968	264.1409, 234.0936, 221.0849, 220.0781, 194.0983	C_18_H_17_NO	Girinimbine	PubChem
40	55.917	286	537.4357, 445.3426, 444.3860, 399.3277, 287.2399, 281.3251	537.4367, 445.3420, 444.3885, 399.3213, 281.3202, 255.1976	C_15_H_10_O_6_	Kaempferol	Standard
41	56.227	–	–	–	–	metabolite	
42	56.882	–	–	–	–	metabolite	
43	57.071	–	–	–	–	metabolite	
44	57.934	–	–	–	–	metabolite	
45	59.277	–	–	–	–	metabolite	
46	66.535	–	–	–	–	metabolite	
47	33.448	190	192.1381, 191.0981, 179.0275	192.1382, 191.1065, 179.0222, 119.0492	C_12_H_14_O_2_	(*Z*)-Ligustilide	Standard
48	74.691	1079	945.5441, 783.4966, 621.4382	1077.5851,945.5450,783.4920, 621.4370, 191.0559	C_53_H_90_O_22_	Ginsenoside Rc	PubChem
49	75.321	638	639.8723, 459.3394 ,441.3280, 424.3384	639, 459, 441, 424	C_36_H_62_O_9_	Ginsenoside F1	PubChem

### Network pharmacology results

#### Prediction and analysis of the target genes

**Figure 2:**
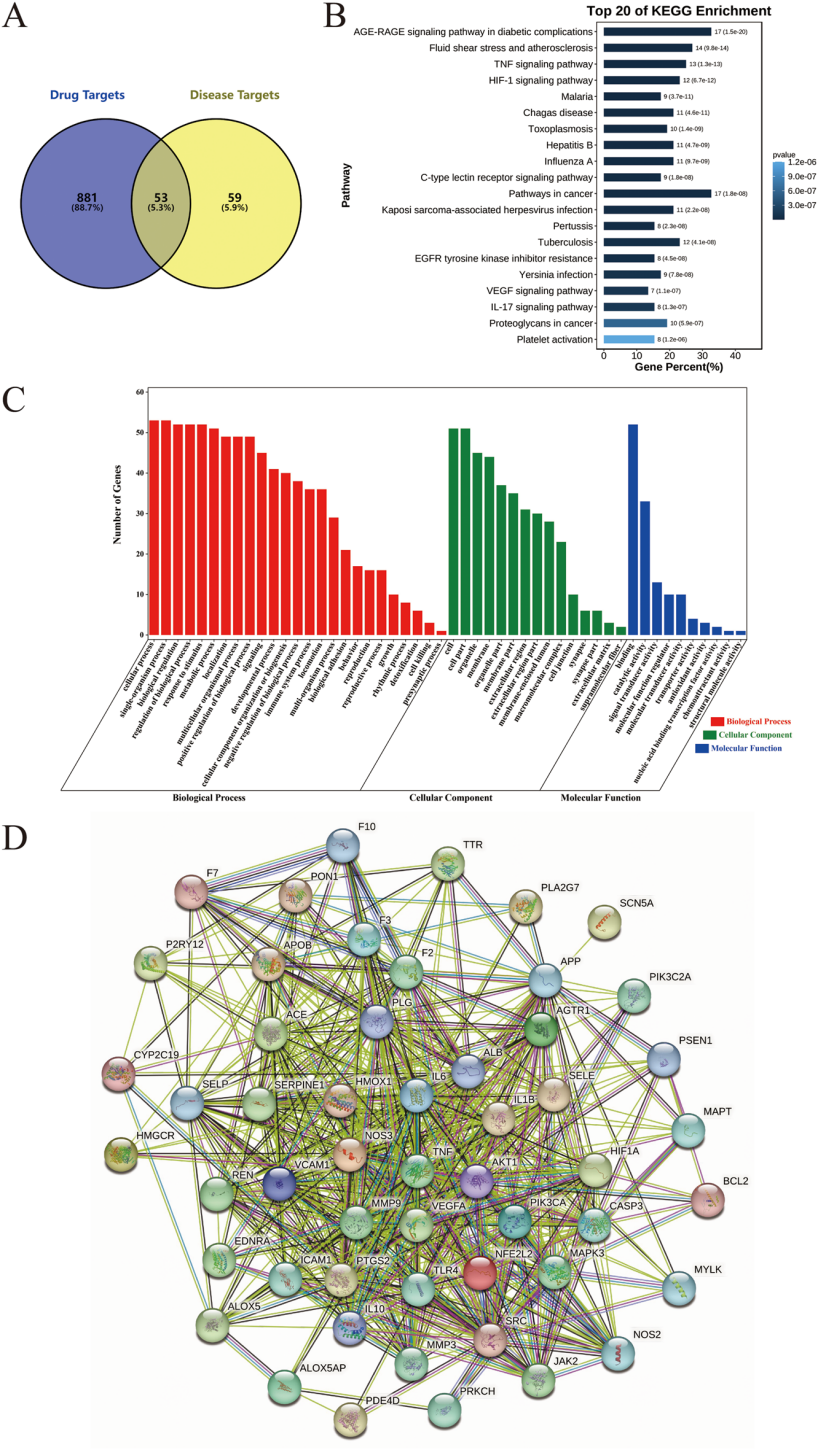
The results of network pharmacology. (**A**) Venn diagram of drug targets and disease targets. (**B**) Top 20 signaling pathways of KEGG pathway enrichment; The smaller the P-value, the more credible the result; The longer the bar, the greater the number of intersection targets enriched. (**C**) GO enrichment; Red represents BP, green represents CC, and blue represents MF; The higher the bar, the greater the number of intersection targets enriched. (**D**) PPI diagram; the more interaction the target, the closer to the center.

A total of 934 targets of incoming blood prototype components and 112 potential targets of IS were obtained through databases. Using Venn diagram to analyze and compare the targets of incoming blood prototype components and IS, a total of 53 intersection targets were obtained, which could be considered as potential targets of NADP in preventing and treating IS, as shown in Fig. 2A. The description of these intersection targets was shown in supplementary Table 1.

The results of KEGG signaling pathway enrichment analysis (Fig. 2B) showed that 52 of the 53 intersection targets were enriched (98.1%), involved in 206 signaling pathways, of which 130 signaling pathways were significantly correlated with target genes (*P* ≤ 0.05). The following pathways involved in the highest participation of targets: AGE-RAGE signaling pathway in diabetic complications (17 targets, 32.69%), such as NOS3, TNF, PIK3CA, AKT1, et al. Pathways in cancer (17 targets, 32.69%), such as NOS2, TNF, PIK3CA, AKT1, HMOX1, NFE2L2, et al. Fluid shear stress and atherosclerosis (14 targets, 26.92%), such as NOS3, TNF, PIK3CA, AKT1, HMOX1, NFE2L2 et al.

The results of GO function enrichment analysis showed that all the 53 intersection targets were enriched (100%), as shown in Fig. 2C. Biological process (BP) enrichment mostly involved in cellular process (53, 100%), single-organism process (53, 100%), regulation of biological process (52, 98.11%), response to stimulus (52, 98.11%) and biological regulation (52, 98.11%). Cell component (CC) enrichment mainly involved in cell (51, 96.23%), cell part (51, 96.23%), organelle (45, 84.91%) and membrane (44, 83.02%). And molecular function (MF) enrichment mainly involved in Binding (52, 98.11%), catalytic activity (33, 62.26%), signal transducer activity (13, 24.528%) and molecular function regulator (10, 18.87%).

The KEGG signaling pathway and GO function enrichment of 53 intersection targets might be the potential mechanism of NADP in preventing and treating IS.

### Analysis of PPI and C-T-P network

The 53 intersection targets were imported into the STRING database to construct the PPI network, then the network was analyzed using Cytoscape 3.9.1 software. The results were shown in Table 3 and Fig. 2D. When there was an interaction between two proteins, it was represented by a connecting line. The more lines, the stronger the interaction ability of protein and the higher the Degree value.

**Table 3:** The Degree score of PPI and C-T-P network.

PPI		C-T-P			
Gene	Degree	Component	Degree	Gene	Degree
TNF	44	Quercetin	26	AKT1	38
NOS3	44	Ginsenoside F1	18	PIK3CA	35
AKT1	42	Ferulic acid	17	PTGS2	29
VEGFA	41	Caffeic acid	16	F2	29
IL6	38	Hydroxysafflor yellow A	15	NOS2	27
HMOX1	36	Folic acid	14	HMOX1	24
IL1B	35	Ginsenoside Rf	14	MAPK3	24
PIK3CA	34	Kaempferol	14	SRC	22
NFE2L2	34	Linoleic Acid	14	TNF	21
MMP9	34	Ginsenoside Rg1	13	NFE2L2	21
ALB	34	Ginsenoside Ro	13	VEGFA	21
ACE	33	Baicalein	13	MMP9	20
MAPK3	33	Apigenin	12	NOS3	20
PTGS2	33	Sinapic acid	12	CASP3	19
SRC	32	Chlorogenic acid	11	APP	16
F2	32	Ginsenoside Rb1	11	ALOX5	16
PLG	32	Ginsenoside Rc	11	IL6	16
TLR4	31	Ginsenoside Rd	11	JAK2	15
CASP3	31	Ginsenoside Re	11	TLR4	14
IL10	30	Schisantherin B	11	IL1B	13

**Figure 3:**
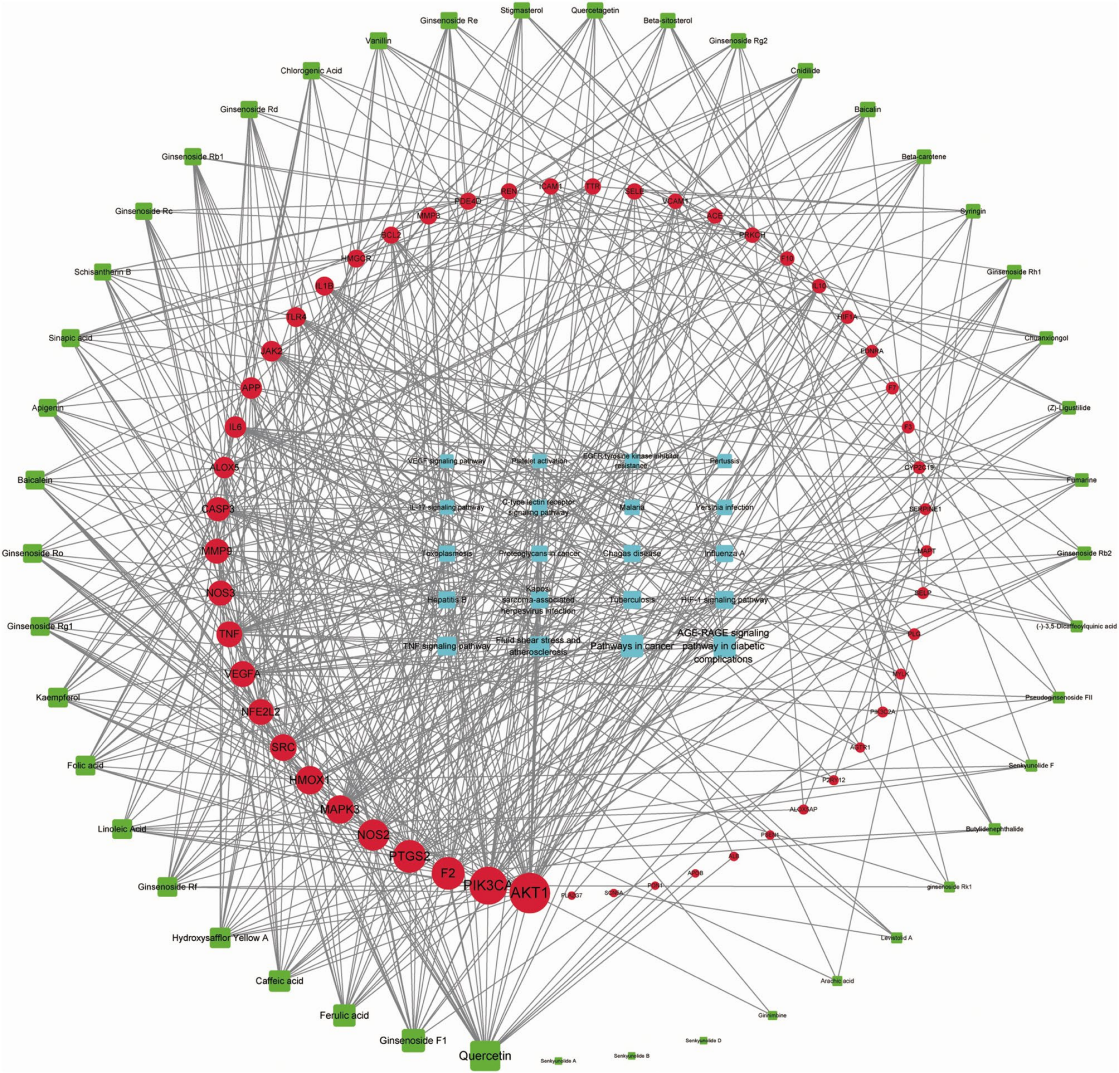
The "C-T-P" network diagram. The blue nodes represent incoming blood components, red nodes represent intersection targets, and green nodes represent signaling pathways. The denser the connection line, the higher the degree.

All the screened data were imported into Cytoscape 3.9.1 software to construct the "C-T-P" network, as shown in Table 3 and Fig. 3. The top-ranked key components were screened out according to the Degree value, such as Quercetin, Ginsenoside F1, Ferulic Acid, Caffeic Acid and Hydroxysafflor Yellow A, as well as core targets, like AKT1, PIK3CA, TNF, NOS3, VEGFA, PTGS2, F2, HMOX1, MAPK3 and NFE2L2.

The above results demonstrated the properties of NADP in the prevention and treatment of IS through multi-component, multi-target and multi-pathway.

### The binding activity of components to target proteins

**Figure 4:**
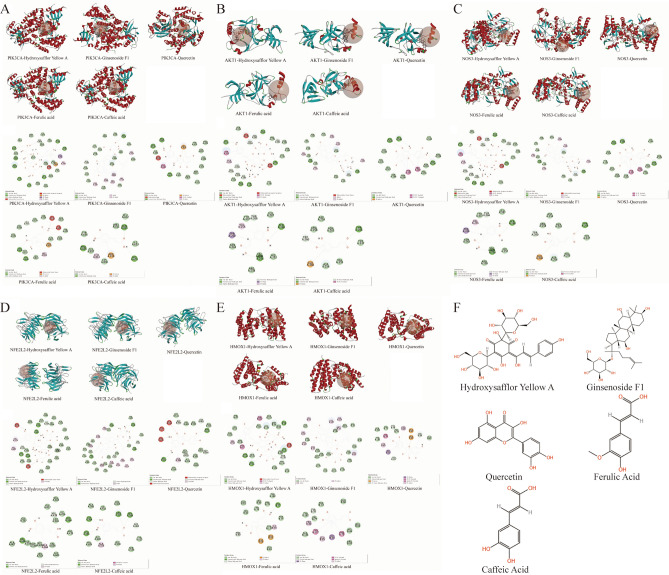
The 3D and 2D high-throughput molecular docking diagram of potential active components and target proteins. (**A**) PIK3CA. (**B**) AKT1. (**C**) NOS3. (**D**) NFE2L2. (**E**) HMOX1. (**F**) Chemical structure of potential active components.

The key components and core targets with high Degree value screened were selected for high-throughput molecular docking, and the molecular docking results were analyzed by LibDock score as screening criterion, as shown in Table 4 and Fig. 4.

**Table 4:** The score results of high-throughput molecular docking.

Target (PDB ID)	Libdock score				
	Hydroxysafflor yellow A	Ginsenoside F1	Quercetin	Ferulic acid	Caffeic acid
PIK3CA(6oac)	160.522	153.593	136.991	107.411	100.365
NFE2L2(7k29)	165.327	158.900	112.965	111.312	108.697
NOS3(1d0c)	147.461	147.982	111.388	108.694	105.762
AKT1(3os5)	134.145	148.797	106.023	108.412	107.132
HMOX1(6eha)	137.990	137.440	132.452	94.861	95.753
F2(5afy)	145.780	140.102	124.150	100.271	79.365
VEGFA(5dn2)	144.251	113.193	111.803	86.942	93.204
MAPK3(4qtb)	139.483	132.073	105.467	93.264	88.907
TNF(5o1e)	138.453	121.980	109.169	93.980	88.720
PTGS2(5f19)	114.485	112.401	113.537	100.182	89.288

It is generally accepted that the higher the docking score, the greater the affinity between the small molecule ligand and large molecule receptor, the stronger the potential binding activity. The results showed that the LibDock score of the core targets PIK3CA, NFE2L2, NOS3, AKT1 and HMOX1 with the key components Hydroxysafflor Yellow A, Ginsenoside F1, Quercetin, Ferulic Acid and Caffeic Acid were all higher than the threshold of 90.0, showing good binding activity.

It was suggested that these five key components in NADP might play an important role in the prevention and treatment of IS, and these five core targets might be the potential targets of NADP in preventing and treating IS.

### In vitro experiments results

#### Effect of NADP on HA1800 cells count

**Figure 5:**
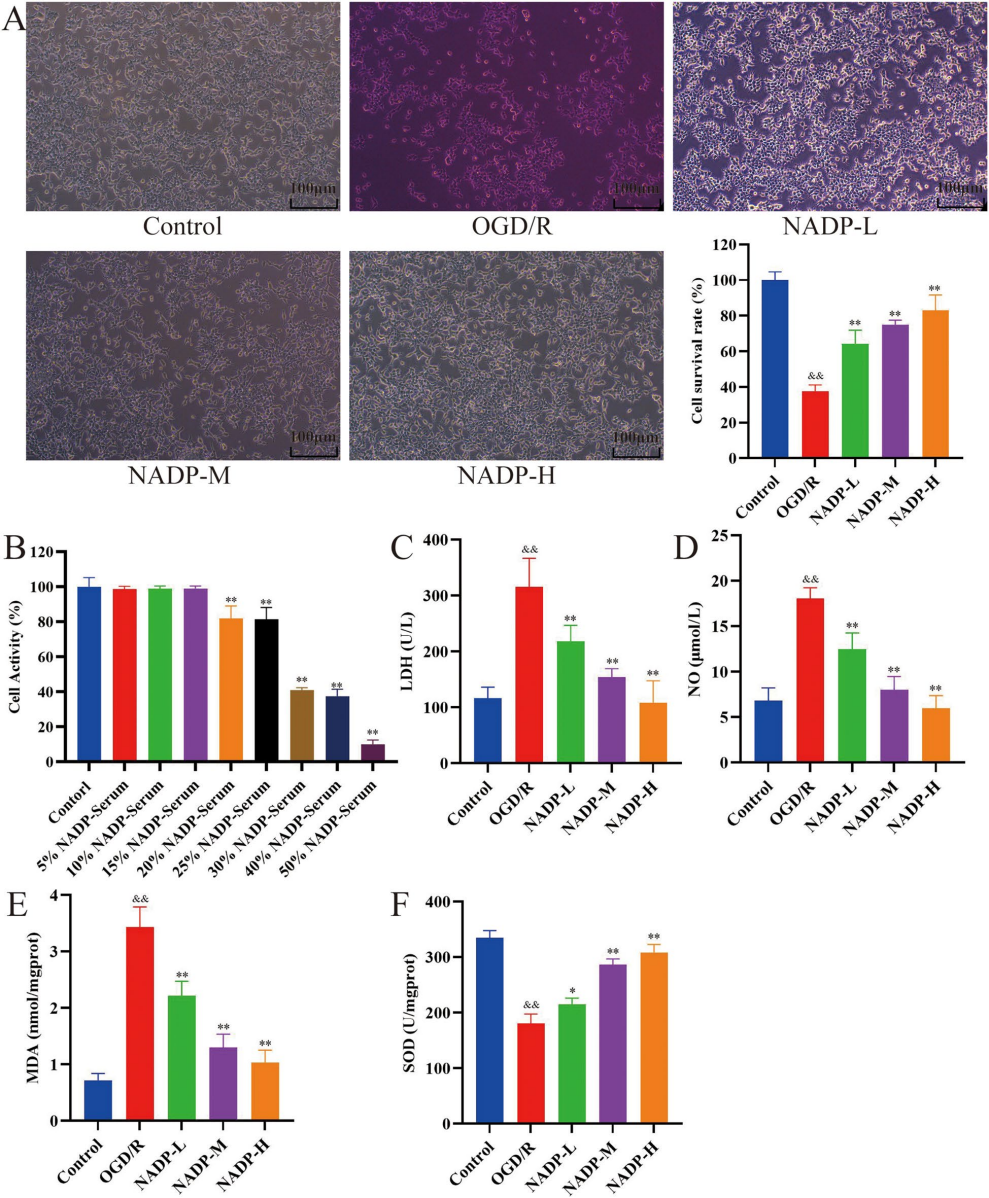
The results of in vitro experiments. (**A**) NADP improved the morphology (100×) and survival rate of HA1800 cells injured by OGD/R (The difference in color was due to the fact that at the same time, the groups had different numbers of cells and consumed different amounts of phenol red-containing medium). (**B**) Effect of NADP on activity of HA1800 cells after 24 h of treatment. (**C**) LDH release. (**D**) NO content. (**E–F**) Content of oxidative stress factors. (**E**) MDA. (**F**) SOD. (Data are mean ± SD; n = 3; Compared with control group, ^&&^*P* < 0.01; Compared with model group, *P < 0.05, **P < 0.01).

As shown in Fig. 5B, the activity of HA1800 cells did not show significant changes in the proportion of 5%, 10%, and 15% NADP-containing serum. When the proportion of NADP-containing serum exceeded 20%, the cell activity decreased significantly (*P* < 0.01), suggesting that at this time NADP exerted a toxic or inhibitory effect on the activity of HA1800 cells. Therefore, the administration concentration of NADP was set at 5% for low, 10% for medium, and 15% for high.

#### Effect of NADP on OGD/R-induced injury to HA1800 cells

After 24 h of OGD/R, the HA1800 cell morphology of each group was observed, as shown in Fig. 5A. Compared with the control group, the number of cells in the model group decreased abruptly, the cell morphology changed from spindle to round, the cell lumen was damaged, and the cell synapses were shortened or even broken. There was a dose-dependent increase in the number of cells in the administered group, and the cell morphology gradually returned to spindle shape, with smooth and shiny cell lumen, extended and longer cell synapses, and obvious intercellular synapses.

The cell survival rate in each group was detected by *MTT* mean, as shown in Fig. 5A. Compared with the control group, the cell survival rate of the model group decreased abruptly (*P* < 0.01), suggesting that OGD/R could damage HA1800 cells and lead to their massive apoptosis. The results showed that NADP ameliorated OGD/R damage to cells in a dose-dependent manner (*P* < 0.01).

#### Effect of NADP on LDH release, NO and oxidative stress factors content

Compared with the control group, LDH release, NO and MDA content were significantly increased (*P* < 0.01) and SOD content was significantly decreased (*P* < 0.01) in the model group, suggesting that OGD/R could lead to HA1800 cellular damage, as shown in Fig. 5C–F. The results showed that NADP were able to dose-dependently inhibit LDH release, NO and MDA production and promote SOD production (*P* < 0.05).

### In vivo experiments results

#### Analysis of neurological symptoms and behavioral in rats

**Figure 6:**
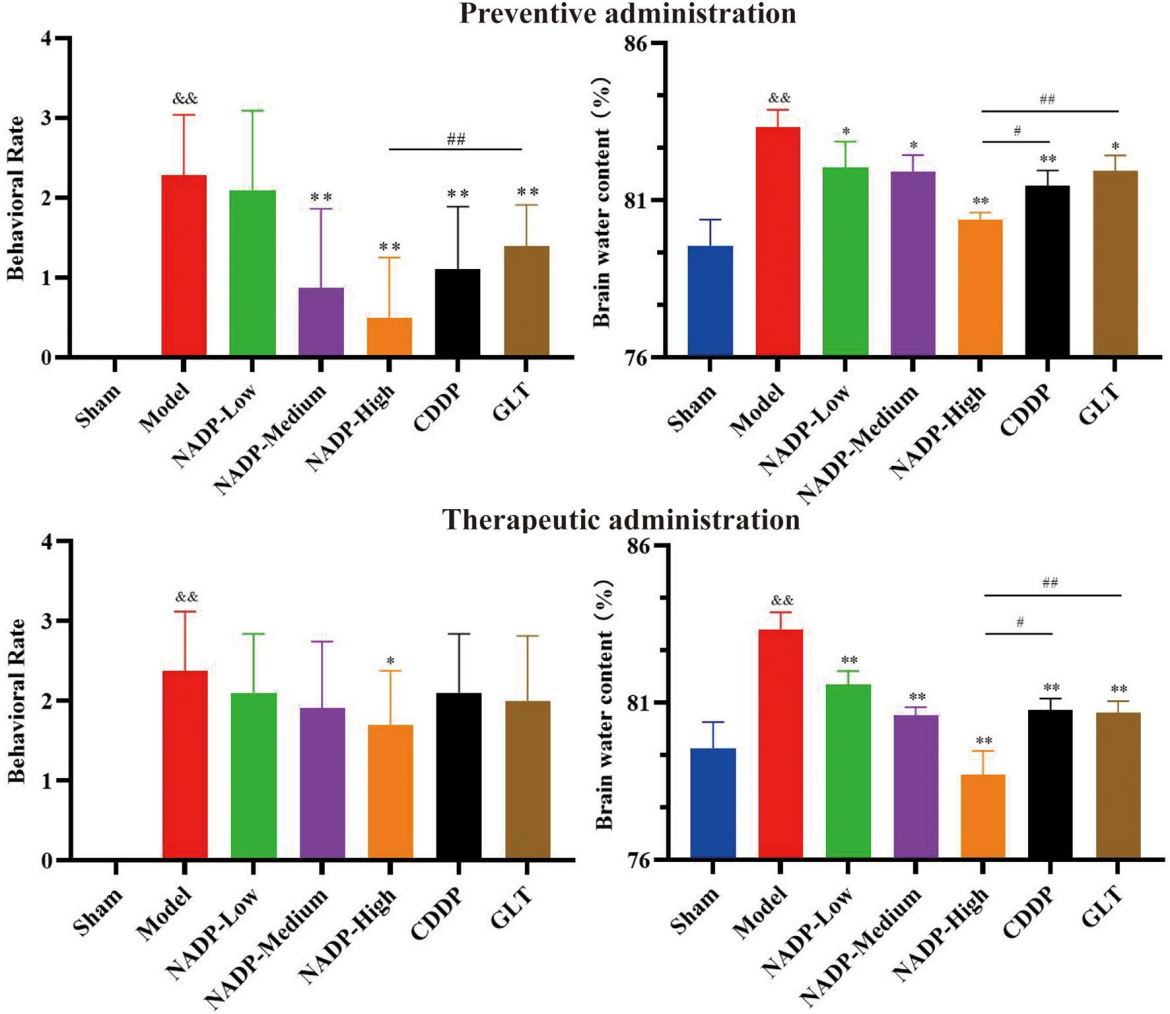
Behavioral rate (n = 10) and brain water content (n = 3) of rats (Data are mean ± SD; Compared with sham group, ^&&^*P* < 0.01; Compared with model group, *P < 0.05, **P < 0.01; Compared with NADP group, ^#^*P* < 0.05, ^##^*P* < 0.01).

The behavior of rats in each group was scored using Zea Longa scoring mean. and the results were shown in Fig. 6 after excluding invalid data (4 pts).

*Preventive administration*. The results showed that NADP was able to dose-dependently improve the neurological symptoms of SD rats, and the improvement effect of NADP-High group on neurological symptoms was significantly stronger than that of GLT group (*P* < 0.01).

*Therapeutic administration*. The results showed that NADP was able to dose-dependently improve the neurological symptoms of SD rats, but only the NADP-High group showed significant difference (*P* < 0.05).

#### Effect of NADP on reduction of brain water content

The brain water content was calculated by weighing the wet and dry weights of brain tissue in each group, as shown in Fig. 6. Compared with the sham group, the brain water content was significantly higher in the model group (*P* < 0.01), suggesting that MCAO/R could lead to cerebral edema.

*Preventive/Therapeutic administration*. The results showed that NADP was able to significantly improve cerebral edema in a dose-dependent manner (*P* < 0.05), and the improvement effect of NADP-High group on cerebral edema was significantly stronger than that of CDDP group (*P* < 0.05) and GLT group (*P* < 0.01), whether preventive administration or therapeutic administration.

#### Effect of NADP on MCAO/R-induced injury to cerebral cortex

**Figure 7:**
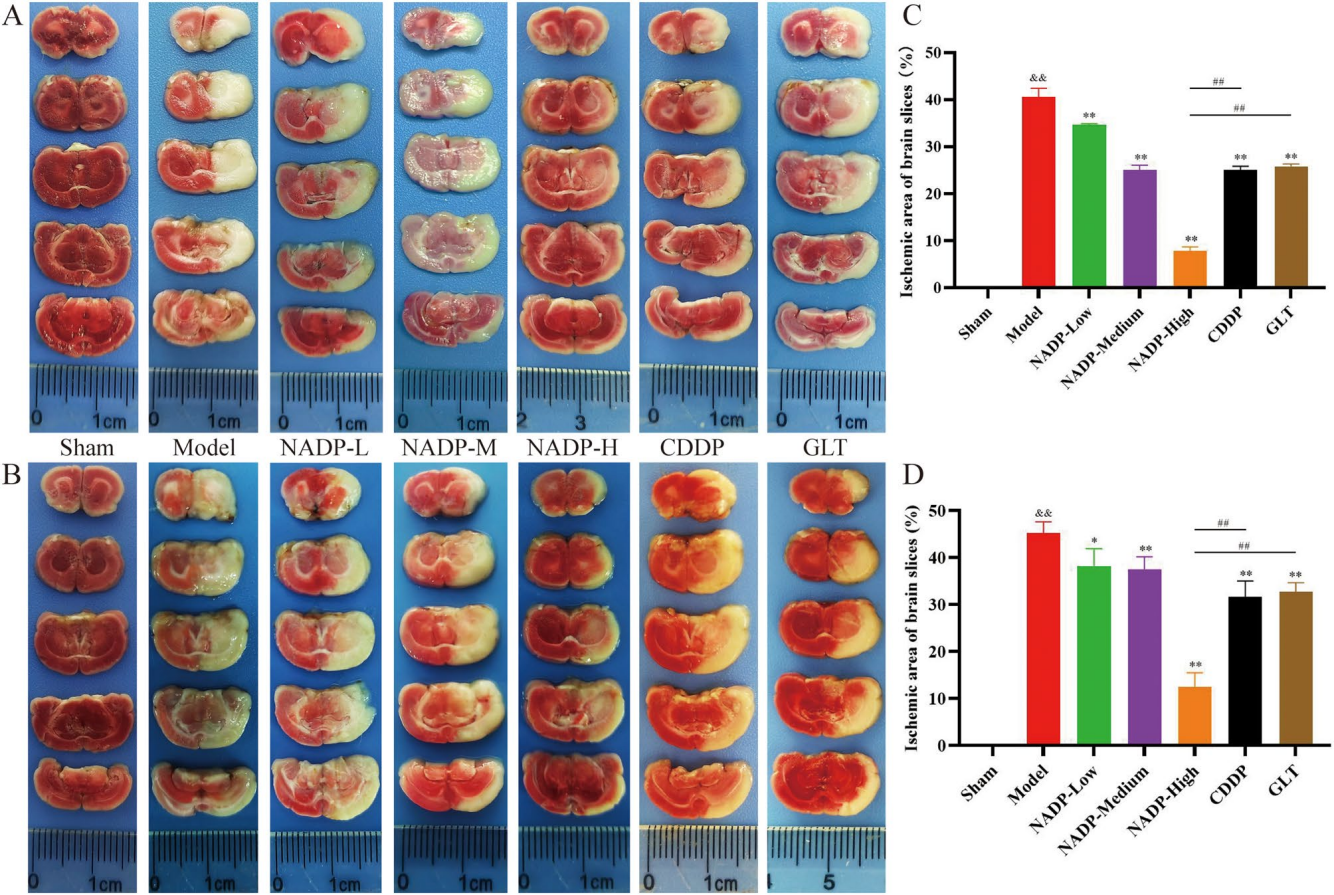
(**A, B**) Comparison of ischemic area of cerebral cortex staining of brain slices (Due to the specificity of MCAO/R, the groups were not performed at the same time, so the images were recorded separately. To ensure the consistency of each group, the proportions were calculated by removing the background and counting only the ischemic area and the total area of the brain slice were calculated). (**C**, **D**) Proportion of ischemic area of cerebral cortex. (**A**/**C**) Preventive administration. (**B**/**D**) Therapeutic administration. (Data are mean ± SD; n = 4; Compared with sham group, ^&&^*P* < 0.01; Compared with model group, *P < 0.05, **P < 0.01; Compared with NADP group, ^##^*P* < 0.01).

The results of TTC staining for ischemic area of cerebral cortex in each group were shown in Fig. 7. Compared with the sham group, the brain tissues of the MCAO/R group and administered group showed obvious white ischemic areas, suggesting that the success of the model and the presence of ischemic area of cerebral cortex.

*Preventive/Therapeutic administration*. The results showed that NADP was able to significantly improve cerebral ischemia due to MCAO/R in a dose-dependent manner (*P* < 0.05), and the improvement effect of NADP-High group on cerebral ischemia was significantly stronger than that of CDDP group and GLT group (*P* < 0.01), whether preventive administration or therapeutic administration.

### Pathological examination of ischemic area of cerebral cortex

**Figure 8:**
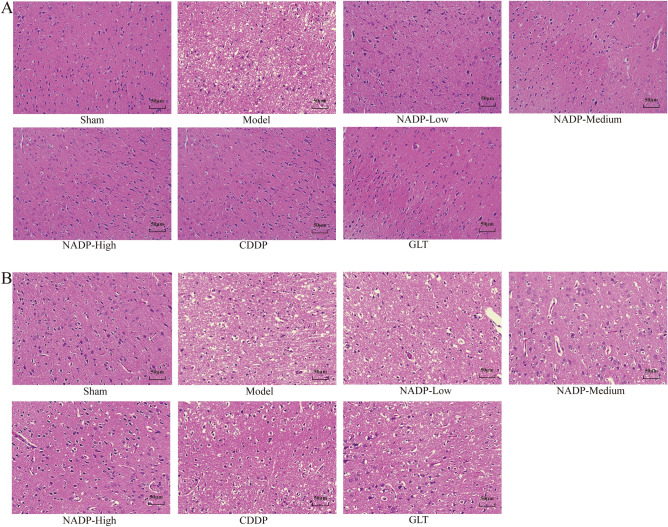
The H&E staining of ischemic area of cerebral cortex (200×, n = 3). (**A**) Preventive administration. (**B**) Therapeutic administration.

Comparison of H&E staining in cerebral cortex in each group was shown in Fig. 8. In the sham group, the cerebral cortex of the brain tissue was structurally normal, the neurocyte was neatly arranged, complete and abundant, the nuclei were clear, large and round, and the cytoplasm was shallow and uniform. In the model group, the cerebral cortex on the ischemic side showed loose reticular structure, with disorganized arrangement, disrupted integrity or even atrophy, reduced number of neurocyte, and fragmented nuclei showed vacuolar necrosis.

*Preventive administration*. The results showed that the pathological damage in the cerebral cortex on the ischemic side of brain tissue was significantly reduced in a dose-dependent manner in NADP administered group, and the morphology of neuronal atrophy was improved, the number of neurocyte increased, and the nuclei were clear. Compared with the CDDP and GLT groups, the structure of cerebral cortex in the NADP-High group was more normal, the neurocyte was neatly arranged and intact, and the nuclear morphology was more normal.

*Therapeutic administration*. The results showed that the pathological damage in the cerebral cortex on the ischemic side of brain tissue was gradually reduced, the arrangement of neurocyte was gradually ordered, and the nuclear vacuoles were gradually reduced in a dose-dependent manner in NADP administered group. Compared with the CDDP and GLT groups, the structure of cerebral cortex in the NADP-High and NADP-Medium groups were more normal, the neurocyte was neatly arranged and intact, and the nuclear morphology was more normal.

### Effect of NADP on Glu content

**Figure 9:**
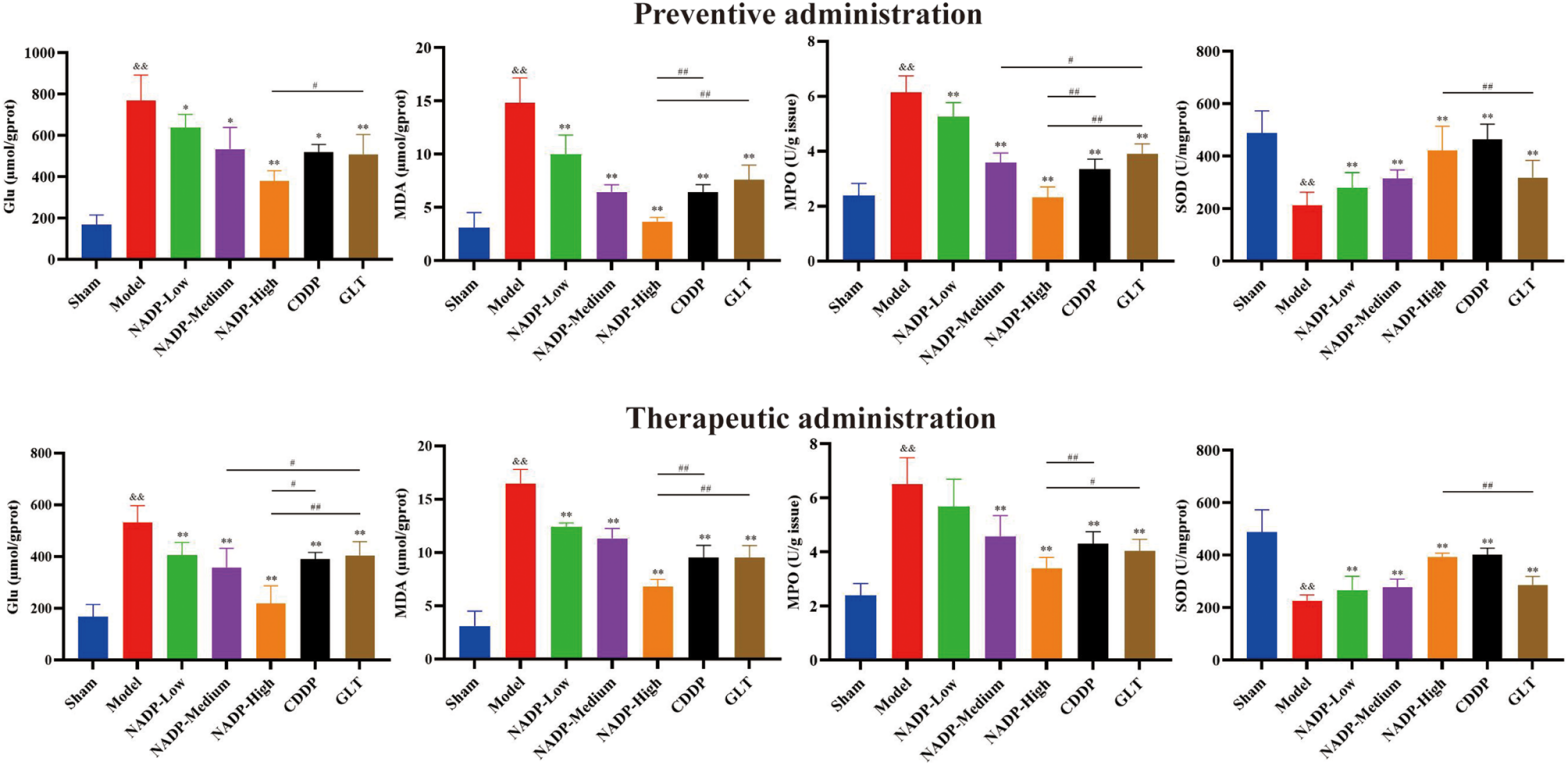
Content of glutamic acid and oxidative stress factors in ischemic area of cerebral cortex in rats. (Data are mean ± SD; n = 3; Compared with sham group, ^&&^*P* < 0.01; Compared with model group, *P < 0.05, **P < 0.01; Compared with NADP group, ^#^*P* < 0.05, ^##^*P* < 0.01).

After the protein concentration examined by BCA kit, Glu content in ischemic area of cerebral cortex in each group was examined by Glu kit, as shown in Fig. 9. Compared with the sham group, the Glu content in ischemic area of cerebral cortex in MCAO/R group increased significantly (*P* < 0.01), suggesting MCAO/R could lead to the increase of excitatory amino acid content and excitatory amino acid toxicity.

*Preventive administration*. The results showed that NADP was able to significantly reduce Glu content in a dose-dependent manner (*P* < 0.05), and the improvement of excitatory amino acid toxicity in the NADP-High group was significantly stronger than that in GLT group (*P* < 0.05).

*Therapeutic administration*. The results showed that NADP was able to significantly reduce Glu content in a dose-dependent manner (*P* < 0.01). The improvement effect on excitatory amino acid toxicity was significantly stronger in the NADP-Medium group than that in GLT group (*P* < 0.05), and the improvement effect on excitatory amino acid toxicity in the NADP-High group was significantly stronger than that in CDDP group (*P* < 0.05) and GLT group (*P* < 0.01).

### Effect of NADP on oxidative stress factors content

The effects of NADP on content of oxidative stress-related factors MDA, MPO and SOD in cerebral cortex on the ischemic side of the brains were shown in Fig. 9. Compared with the sham group, the MDA and MPO content in cerebral cortex in MCAO/R group increased significantly (*P* < 0.01), while the SOD content increased significantly (*P* < 0.01), suggesting MCAO/R could lead to oxidative stress.

*Preventive/Therapeutic administration*. The results showed that NADP was able to significantly reduce MDA and MPO content (*P* < 0.01), and increase SOD content in a dose-dependent manner (*P* < 0.01), and the improvement effect of NADP-High group on oxidative stress was stronger than that in CDPP group and GLT group, whether preventive administration or therapeutic administration.

#### Effect of NADP on PI3K/AKT/eNOS in ischemic area of cerebral cortex

**Figure 10:**
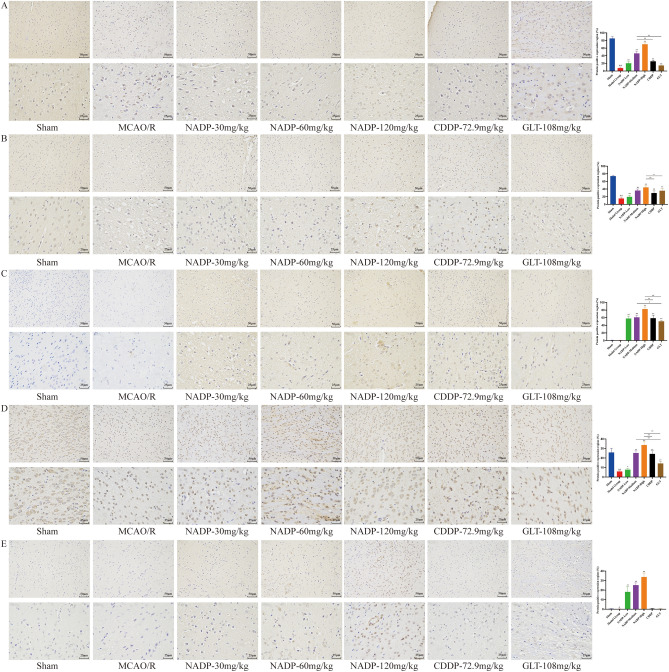
Images of ICH for preventive administration on PI3K/AKT/eNOS in ischemic area of cerebral cortex in rats (200×, 400×). (**A**) PI3K. (**B**) Akt. (**C**) p-Akt-S437. (**D**) eNOS. (**E**) p-eNOS-S1177. (Data are mean ± SD; n = 3; Compared with sham group, ^&&^*P* < 0.01; Compared with model group, *P < 0.05, **P < 0.01; Compared with NADP group, ^#^*P* < 0.05, ^##^*P* < 0.01).

**Figure 11:**
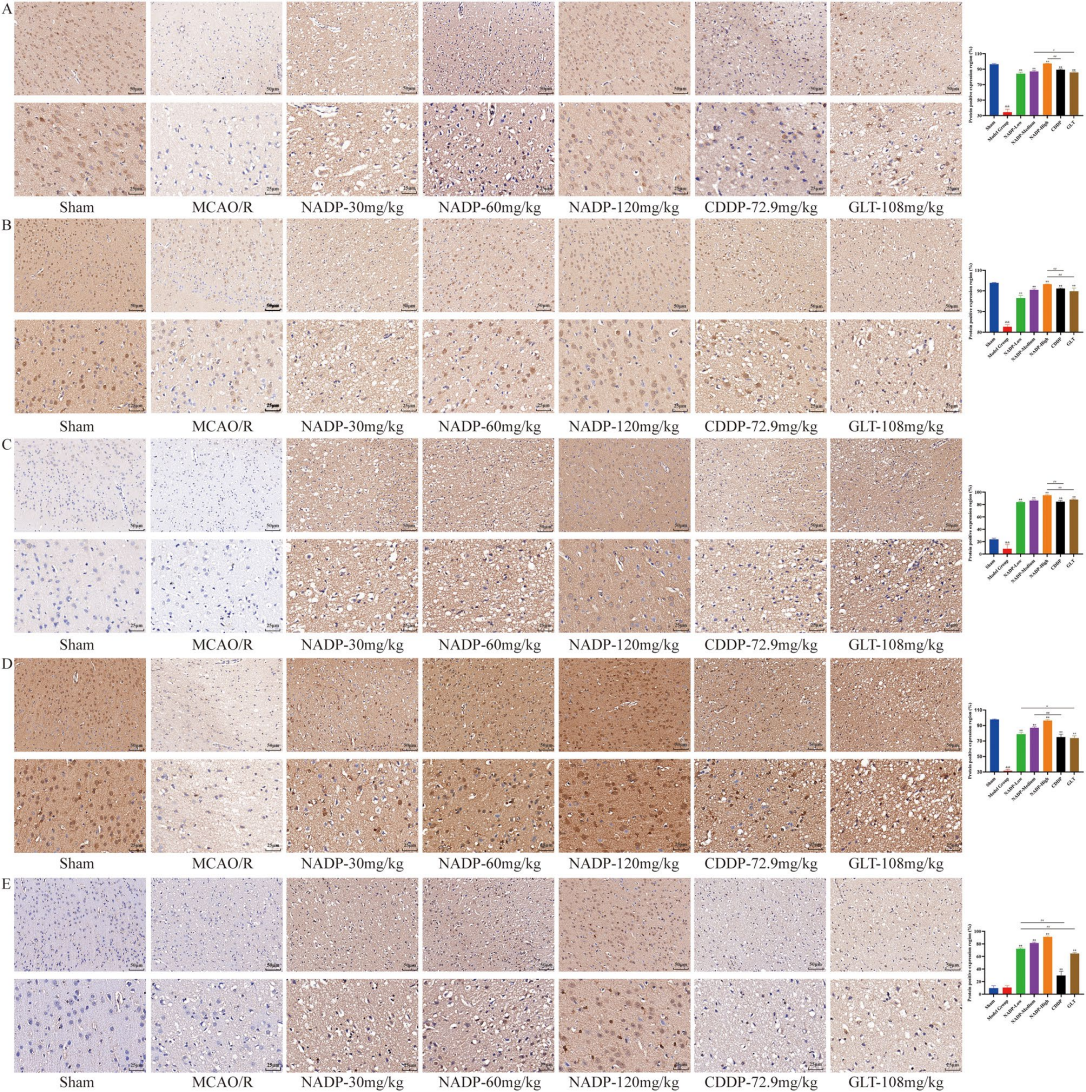
Images of ICH for therapeutic administration on PI3K/AKT/eNOS in ischemic area of cerebral cortex in rats (200×, 400×). (**A**) PI3K. (**B**) Akt. (**C**) p-Akt-S437. (**D**) eNOS. (**E**) p-eNOS-S1177. (Data are mean ± SD; n = 3; Compared with sham group, ^&&^*P* < 0.01; Compared with model group, **P < 0.01; Compared with NADP group, ^#^*P* < 0.05, ^##^*P* < 0.01).

The effect of NADP on the expression of PI3K/AKT/eNOS were shown in Fig. 10 and Fig. 11 (proteins were stained yellowish-brown).

*Preventive administration*. Compared with the sham group, the expression of PI3K, Akt, eNOS and p-eNOS-S1177 in cerebral cortex of MCAO/R group decreased significantly (*P* < 0.05). The results showed that NADP was able to significantly promote the expression of PI3K, Akt, p-Akt-S473, eNOS and p-eNOS-S1177 in a dose-dependent manner (*P* < 0.05), while both CDDP and GLT could also significantly promote the expression of PI3K, Akt, p-Akt-S473 and eNOS (*P* < 0.01) but no significant change of p-eNOS-S1177. The effects of NADP-Medium on promoting the expression of PI3K and p-eNOS-S1177 were better than that of CDDP (*P* < 0.01), while promoting the expression of PI3K, p-Akt-S473, eNOS and p-eNOS-S1177 were better than that of GLT (*P* < 0.05). The effects of NADP-High on promoting the expression of PI3K, Akt, p-Akt-S473, eNOS and p-eNOS-S1177 were significantly better than that of CDDP (*P* < 0.01) and GLT (*P* < 0.05).

*Therapeutic administration*. Compared with the sham group, the expression of PI3K, Akt, p-Akt and eNOS in cerebral cortex of MCAO/R group decreased significantly (*P* < 0.01). The results showed that NADP, CDDP and GLT were able to significantly promote the expression of PI3K, Akt, p-Akt-S473, eNOS and p-eNOS-S1177 in a dose-dependent manner (*P* < 0.01). The effect of NADP-Low on promoting the expression of p-eNOS-S1177 was better than that of CDDP (*P* < 0.01), while promoting the expression of eNOS and p-eNOS-S1177 was better than that of GLT (*P* < 0.05). The effect of NADP-Medium on promoting the expression of eNOS was better than that of CDDP (*P* < 0.01), while promoting the expression of PI3K was better than that of GLT (*P* < 0.05). The effects of NADP-High on promoting the expression of PI3K, Akt and p-Akt-S473 were better than that of CDDP (*P* < 0.01), while promoting the expression of Akt and p-Akt-S473 were better than that of GLT (*P* < 0.01).

#### Effect of NADP on Nrf2/HO-1 in ischemic area of cerebral cortex

**Figure 12:**
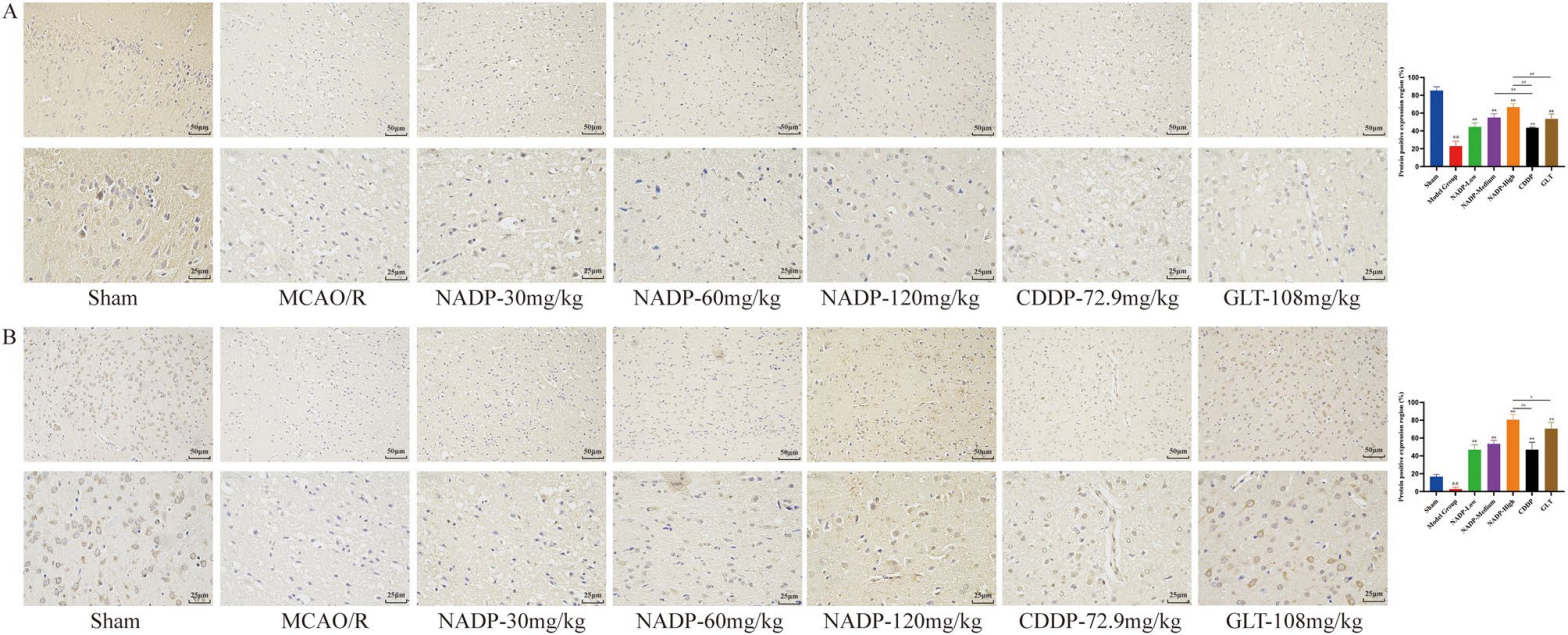
Images of ICH for preventive administration on Nrf2/HO-1 in ischemic area of cerebral cortex in rats (200×, 400×). (**A**) Nrf2. (**B**) HO-1. (Data are mean ± SD; n = 3; Compared with sham group, ^&&^*P* < 0.01; Compared with model group, **P < 0.01; Compared with NADP group, ^#^*P* < 0.05, ^##^*P* < 0.01).

**Figure 13:**
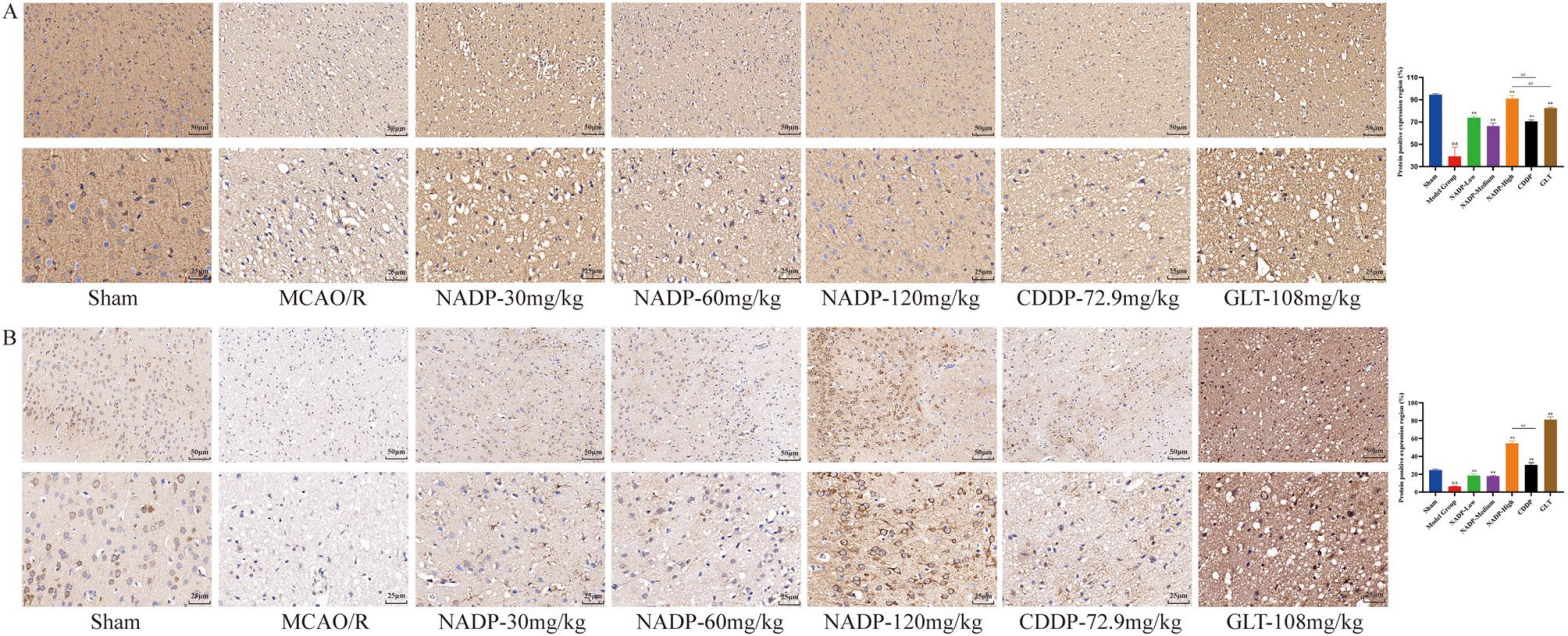
Images of ICH for therapeutic administration on Nrf2/HO-1 in ischemic area of cerebral cortex in rats (200×, 400×). (**A**) Nrf2. (**B**) HO-1. (Data are mean ± SD; n = 3; Compared with sham group, ^&&^*P* < 0.01; Compared with model group, **P < 0.01; Compared with NADP group, ^##^*P* < 0.01).

The effect of NADP on the expression of PI3K/AKT/eNOS were shown in Fig. 12 and Fig. 13 (proteins were stained yellowish-brown).

*Preventive administration*. Compared with the sham group, the expression of Nrf2 and HO-1 in cerebral cortex of MCAO/R group decreased significantly (*P* < 0.01). The results showed that NADP, CDDP and GLT were able to significantly promote the expression of Nrf2 and HO-1 in a dose-dependent manner (*P* < 0.01). The effect of NADP-Medium on promoting the expression of Nrf2 was better than that of CDDP (*P* < 0.01). The effects of NADP-High on promoting the expression of Nrf2 and HO-1 were better than that of CDDP (*P* < 0.01) and GLT (*P* < 0.05).

*Therapeutic administration*. Compared with the sham group, the expression of Nrf2 and HO-1 in cerebral cortex of MCAO/R group decreased significantly (*P* < 0.01). The results showed that NADP, CDDP and GLT were able to significantly promote the expression of Nrf2 and HO-1 (*P* < 0.01). The effects of NADP-High on promoting the expression of Nrf2 and HO-1 were better than that of CDDP (*P* < 0.01), while promoting the expression of Nrf2 was better than that of GLT (*P* < 0.01).

## Discussion

As a commonly used secondary prevention drug for IS in clinic, NADP has unique and proven efficacy. However, the complexity of traditional Chinese medicine and its compound prescription has led to the lack of clarity about the material basis and mechanism of NADP in the prevention and treatment of IS. In this study, UHPLC-MS/MS mean was used to analyze the incoming blood components of NADP administration of rat serum, and using network pharmacology and high-throughput molecular docking to predict the key bioactive components and core targets which might be the important information of NADP in preventing and treating IS, then the efficacy and mechanism of NADP in preventing and treating IS were explored and verified by in vivo and in vitro experiments.

A total of 45 incoming blood prototype components were identified from NADP-containing rat serum by UHPLC-MS/MS analysis. It was reported that Caffeic Acid played the role in reducing neuroinflammation and resisting ferroptosis through the Nrf2 signaling pathway, suggesting that caffeic acid might be a potential therapeutic method for alleviating brain injury after cerebral ischemia^27^. Studies have shown that Hydroxysafflor Yellow A, the most pharmacologically active chalcone water-soluble component in *Carthamus tinctorius* L., could prevent IS by anti-coagulant, cerebral vasodilatation and improvement of cerebral vascular permeability^28^. Meanwhile, Hydroxysafflor Yellow A may act as a post-IS neuroprotective agent by inhibiting autophagy induction by inhibiting HIF-1, BNIP3 and Notch1^29^. (*Z*)-Ligustilide from *Ligusticum chuanxiong* Hort. and *Angelica sinensis* (Oliv.) Diels has been reported to protect nerve damage against IS in vivo and in vitro by inducing Drp1-mediated mitochondrial fission via activation of AMPK signaling pathway^30^. The 45 incoming blood prototype components might be the material basis for the efficacy of NADP.

The results of network pharmacology showed that the potential active components of NADP, Hydroxysafflor Yellow A, Ginsenoside F1, Quercetin, Ferulic Acid and Caffeic Acid, might bind to organelles and cell membranes to regulate the expression of PIK3CA, NFE2L2, NOS3, AKT1 and HMOX1, catalyze the synthesis of NO, thus inhibiting platelet activation, aggregation and inflammation, inhibiting thrombosis and oxidative stress, to prevent and treat IS. It suggested that the incoming blood prototype components of NADP were able to prevent and treat IS through multi-targets and multi-pathways. However, due to the limitations of the network pharmacology, there may be some discrepancies between the predicted mechanism and the results of actual basic research, so it is necessary to further verify the results of network pharmacology.

The PI3K/Akt signaling pathway is one of the most important intracellular signaling transduction pathways, which mediates cell migration, proliferation, differentiation and apoptosis by affecting the activation of downstream effector molecules and plays an important role in physiological or pathophysiological conditions^31^. Nitric oxide synthase has been classified into three subtypes according to the regulation of prototype enzyme expression, cell or tissue origin, and order of cloning: neuronal NOS, endothelial NOS (eNOS) and inducible-expressing NOS^32^. Among them, eNOS is responsible for synthesizing most of the NO produced in blood vessels, and the absence of eNOS can lead to various pathophysiological disorders including vascular diseases^33^. Ser1177 site phosphorylation has been considered to be one of the most important factors in the regulation of eNOS activity. The regulatory role of PI3K/Akt signaling pathway in neuronal apoptosis after cerebral ischemia is of great interest. With the assistance of [pyruvate dehydrogenase (acetyl-transferring)] kinase isozyme 2, mitochondrial, PI3K enhances the activity of the Akt protein by phosphorylating its Ser473 site, and then phosphorylates the Ser1177 site of eNOS in neurons and endothelial cells, thereby activating the eNOS enzyme^34^. Phosphorylation of Akt and eNOS is an important process in the synthesis of NO by endothelial cells, while moderate amount of NO plays an important role in neuroprotection and vascular protection, but excessive NO is cytotoxic and promotes inflammation and injury through the formation of toxic active nitrogen^35^. A rat cerebral ischemia study showed that phosphorylation of the eNOS Ser1177 site could regulate cerebral blood flow, inhibit apoptosis and improve cerebral ischemic injury^36^. In addition, previous studies have confirmed that PI3K/Akt/eNOS plays an important role in cerebral protection and regulation of brain tissue apoptosis induced by OGD/R and MCAO/R^37,38^.

Oxidative stress is a major factor in endothelial cell activation and dysfunction, whereas apoptosis is the main mechanism of oxidative stress-induced endothelial cell injury. As an indispensable signaling pathway in oxidative stress response, the Nrf2/HO-1 signaling pathway is involved in the anti-inflammatory, antioxidant and apoptosis^39^. The transcription factor Nrf2, which is encoded by the NFE2L2 gene, is a major regulator of cellular and tissue defense against endogenous and exogenous stressors, and achieves stress-induced activation by coordinating the basis of multiple cytoprotective genes^40^. The Nrf2 network not only controls redox homeostasis, but also regulates a variety of intermediary metabolic processes^41^. As a result, targeting Nrf2 has emerged as an attractive therapeutic strategy for the prevention and treatment of central nervous system diseases, including stroke. HO-1 is a type II antioxidant enzyme regulated by Nrf2, which plays a crucial role in the degradation and metabolism of heme^42^. HO-1 can convert hemoglobin to CO, Fe^2+^ and biliverdin, and reduce these products to bilirubin, thereby exerting antioxidant, anti-inflammatory, anti-apoptotic, and anti-thrombotic effects^43^. Therefore, the activation of Nrf2/HO-1 signaling pathway would be beneficial in protecting endothelial cells from oxidative stress-induced injury and inhibiting IS.

In vitro results showed that NADP could inhibit OGD/R-induced apoptosis, suppress LDH release and reduce toxic NO production, and ameliorate the damage induced by OGD/R to HA1800 cells. Meanwhile, NADP was also able to increase the SOD content, decrease the MDA content, and ameliorate the OGD/R-induced oxidative stress damage to HA1800.

In vivo results showed that NADP was able to improve neurological symptoms of rats, reduce the infarct volume of cerebral cortical area and relieve cerebral edema. The pathological results showed that the pathological damage of the cerebral cortex on the ischemic side of brain tissues in NADP administration group was alleviated, the atrophic morphology of neurocyte was improved and increased in number, and the nuclei were clearly, suggesting that NADP had an obvious protective effect on the cerebral cortex on the ischemic side. Meanwhile, NADP could inhibit Glu production, which in turn increase SOD content and decrease MDA and MPO content, so as to ameliorate the excitatory amino acid toxicity and oxidative stress-induced damage to brain tissues.

Immunohistochemical results showed that NADP was able to up-regulate the expression of PI3K, Akt and eNOS, and phosphorylate the S473 site of Akt and the S1177 site of eNOS, promote the synthesis of NO, induce vasodilation, increase cerebral blood flow, inhibit apoptosis of neurocyte and supply substances necessary for metabolism to protect the brain tissues. Meanwhile, NADP was also able to up-regulate the expression of Nrf2, initiate its transcriptional process to regulate the expression of the downstream protein HO-1, reduce the oxidative stress factors content and harmful substances, and promote the anti-oxidative stress and other cytoprotective mechanisms, so as to ameliorate the oxidative stress injury induced by MCAO/R.

There are still deficiencies in the experiments. This study is more focused on the protective effect of NADP on brain tissue, in the serum pharmacochemistry, the components that cross the blood–brain barrier should be analyzed, so the cerebrospinal fluid should be taken for analysis and identification of the incoming blood components. It has been reported that the components in *Ligusticum chuanxiong* Hort. and *Angelica sinensis* (Oliv.) Diels, such as ligustrazine and (*Z*)-Ligustilide, which have strong activities in the prevention and treatment of cerebral ischemia, but they have not been identified from the NADP-containing serum or screened out from network pharmacology, which is where the limitations of serum pharmacochemistry and network pharmacology come into play. Meanwhile, the potential pharmacodynamic substances predicted by network pharmacology have not been further verified in experiments, so the in vivo and in vitro pharmacodynamic and mechanism studies of Hydroxysafflor Yellow A, Ginsenoside F1, Quercetin, Ferulic Acid and Caffeic Acid in preventing and treating IS will be continued in the follow-up.

## Supplementary Information

Supplementary Information.

## Data Availability

The datasets used and/or analyzed during the current study were available from the corresponding author on reasonable request. The main supporting data can be found in the supplementary material.

## Abbreviations

BP: Biological process
CC: Cell component
CCA: Common carotid artery
CDDP: Compound Danshen Dropping Pill
C-T-P: Component-target-pathway
DL: Drug like
ECA: External carotid artery
GLT: Ginkgo leaf tablet
Glu: Glutamate
GO: Gene ontology
HA1800: Normal human brain astrocyte cell
ICA: Internal carotid artery
IS: Ischemic stroke
KEGG: Kyoto encyclopedia of genes and genomes
LDH: Lactate dehydrogenase
MCAO/R: Middle cerebral artery occlusion/reperfusion
MDA: Malondialdehyde
MF: Molecular functions
MPO: Myeloperoxidase
NADP: Nao-an dropping pill
NOS3: Nitric oxide synthase, endothelial
OB: Oral bioavailability
OD: Optical density
OGD/R: Oxygen–glucose deprivation/reperfusion
PPI: Protein–protein interaction
SOD: Superoxide dismutase
TTC: 2,3,5-Triphenyltetrazolium chloride
