# Author Correction: Revealing the pharmacological mechanisms of nao-an dropping pill in preventing and treating ischemic stroke via the PI3K/Akt/eNOS and Nrf2/HO-1 pathways

**DOI:** 10.1038/s41598-025-94461-9

**Published:** 2025-04-08

**Authors:** Chen Wang, Zhe-Ming Xiong, You-Quan Cong, Zi-Yao Li, Yi Xie, Ying-Xiao Wang, Hui-Min Zhou, Yan-Fang Yang, Jing-Jing Liu, He-Zhen Wu

**Affiliations:** 1 College of Pharmacy, Hubei University of Chinese Medicine, Wuhan, 430065 China; 2 Leiyunshang Pharmaceutical Group Co., Ltd, Suzhou, 215009 China; 3 Key Laboratory of Traditional Chinese Medicine Resources and Chemistry of Hubei Province, Wuhan, 430065 China; 4 Modern Engineering Research Center of Traditional Chinese Medicine and Ethnic Medicine of Hubei Province, Wuhan, 430065 China

Correction to: *Scientific Reports* 10.1038/s41598-024-61770-4, published online 16 May 2024

The original version of this Article contained an error in Figure 8 where the images of ‘NADP-High’ and ‘CDDP’ groups were repeated in panel (a). The original Figure 8 and accompanying legend appear below.

**Fig. 8:**
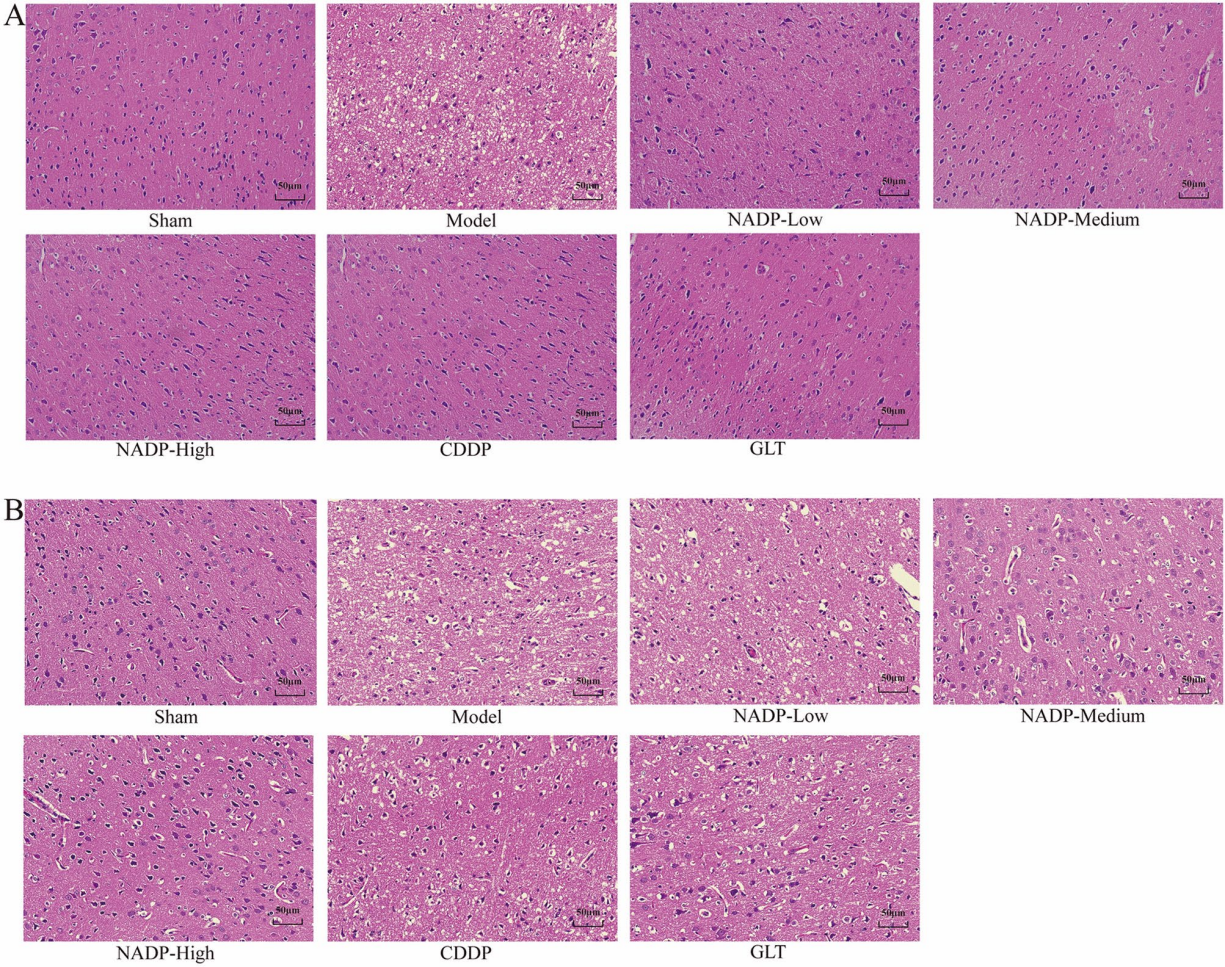
The H&E staining of ischemic area of cerebral cortex (200×, n = 3). (**A**) Preventive administration. (**B**) Therapeutic administration.

The original Article has been corrected.

