# Supplementary material for: Revealing the pharmacological mechanisms of nao-an dropping pill in preventing and treating ischemic stroke via the PI3K/Akt/eNOS and Nrf2/HO-1 pathways
Source: Sci Rep. 2024 May 16;14:11240. doi: 10.1038/s41598-024-61770-4 (PMC11099061; doi:10.1038/s41598-024-61770-4)
Supplement: Supplementary file 1 — Supplementary Information. [file 41598_2024_61770_MOESM1_ESM.docx]

Supplementary Table 1. The information of intersection targets

| **No.** | **Symbol** | **Target** | **Uniprot ID** | **Target Class** |
| --- | --- | --- | --- | --- |
| 1 | ACE | Angiotensin-converting enzyme | P12821 | Protease |
| 2 | AGTR1 | Type-1 angiotensin II receptor | P30556 | protein-coupled receptor |
| 3 | AKT1 | Serine/threonine-protein kinase AKT | P31749 | Kinase |
| 4 | ALB | Serum albumin | P02768 | Secreted protein |
| 5 | ALOX5 | Arachidonate 5-lipoxygenase | P09917 | Oxidoreductase |
| 6 | ALOX5AP | 5-lipoxygenase activating protein | P20292 | Other cytosolic protein |
| 7 | APOB | Apolipoprotein B | P04114 | Secreted protein |
| 8 | APP | Beta amyloid A4 protein | P05067 | Membrane receptor |
| 9 | BCL2 | Apoptosis regulator Bcl-2 | P10415 | Other ion channel |
| 10 | CASP3 | Caspase-3 | P42574 | Protease |
| 11 | CYP2C19 | Cytochrome P450 2C19 | P33261 | Cytochrome P450 |
| 12 | EDNRA | Endothelin receptor ET-A | P25101 | protein-coupled receptor |
| 13 | F10 | Thrombin and coagulation factor X | P00742 | Protease |
| 14 | F2 | Thrombin | P00734 | Protease |
| 15 | F3 | Coagulation factor VII/tissue factor | P13726 | Surface antigen |
| 16 | F7 | Coagulation factor VII | P08709 | Protease |
| 17 | HIF1A | Hypoxia-inducible factor 1 alpha | Q16665 | Transcription factor |
| 18 | HMGCR | HMG-CoA reductase | P04035 | Oxidoreductase |
| 19 | HMOX1 | Heme oxygenase 1 | P09601 | Enzyme |
| 20 | ICAM1 | Intercellular adhesion molecule-1 | P05362 | Adhesion |
| 21 | IL10 | Interleukin-10 | P22301 | Secreted protein |
| 22 | IL1B | Interleukin-1 beta | P01584 | Secreted protein |
| 23 | IL6 | Interleukin-6 | P05231 | Secreted protein |
| 24 | JAK2 | Tyrosine-protein kinase JAK2 | O60674 | Kinase |
| 25 | MAPK3 | MAP kinase ERK1 | P27361 | Kinase |
| 26 | MAPT | Microtubule-associated protein tau | P10636 | Unclassified protein |
| 27 | MMP3 | Matrix metalloproteinase 3 | P08254 | Protease |
| 28 | MMP9 | Matrix metalloproteinase 9 | P14780 | Protease |
| 29 | MYLK | Myosin light chain kinase, smooth muscle | Q15746 | Kinase |
| 30 | NFE2L2 | Nuclear factor erythroid 2-related factor 2 | Q16236 | Unclassified protein |
| 31 | NOS2 | Nitric oxide synthase, inducible | P35228 | Enzyme |
| 32 | NOS3 | Nitric oxide synthase, endothelial | P29474 | Enzyme |
| 33 | P2RY12 | Purinergic receptor P2Y12 | Q9H244 | protein-coupled receptor |
| 34 | PDE4D | Phosphodiesterase 4D | Q08499 | Phosphodiesterase |
| 35 | PIK3C2A | Phosphatidylinositol-4-phosphate 3-kinase C2 domain-containing subunit alpha | O00443 | Enzyme |
| 36 | PIK3CA | PI3-kinase p110-alpha subunit | P42336 | Enzyme |
| 37 | PLA2G7 | LDL-associated phospholipase A2 | Q13093 | Enzyme |
| 38 | PLG | Plasminogen | P00747 | Protease |
| 39 | PON1 | Serum paraoxonase/arylesterase 1 | P27169 | Secreted protein |
| 40 | PRKCH | Protein kinase C eta | P24723 | Kinase |
| 41 | PSEN1 | Presenilin-1 | P49768 | Protease |
| 42 | PTGS2 | Cyclooxygenase-2 | P35354 | Oxidoreductase |
| 43 | REN | Renin | P00797 | Protease |
| 44 | SCN5A | Sodium channel protein type V alpha subunit | Q14524 | Voltage-gated ion channel |
| 45 | SELE | Selectin E | P16581 | Adhesion |
| 46 | SELP | P-selectin | P16109 | Adhesion |
| 47 | SERPINE1 | Plasminogen activator inhibitor-1 | P05121 | Secreted protein |
| 48 | SRC | Tyrosine-protein kinase SRC | P12931 | Kinase |
| 49 | TLR4 | Toll-like receptor 4 | O00206 | Toll-like and Il-1 receptors |
| 50 | TNF | TNF-alpha | P01375 | Secreted protein |
| 51 | TTR | Transthyretin | P02766 | Secreted protein |
| 52 | VCAM1 | Vascular cell adhesion protein 1 | P19320 | Adhesion |
| 53 | VEGFA | Vascular endothelial growth factor A | P15692 | Secreted protein |


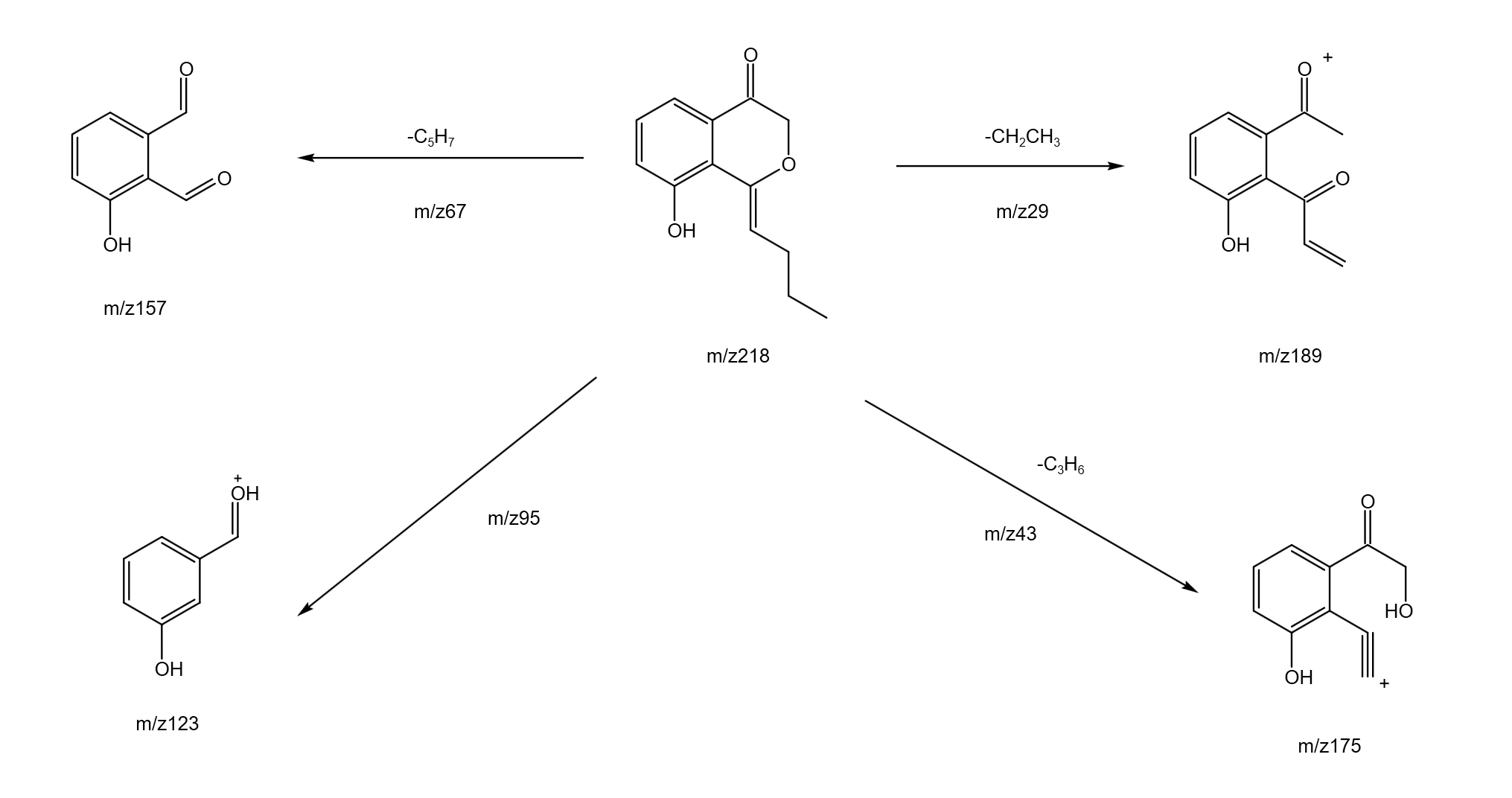


Supplementary Fig. 1 Spectrometry fragmentation modes of Chuanxiongol in positive ion mode.


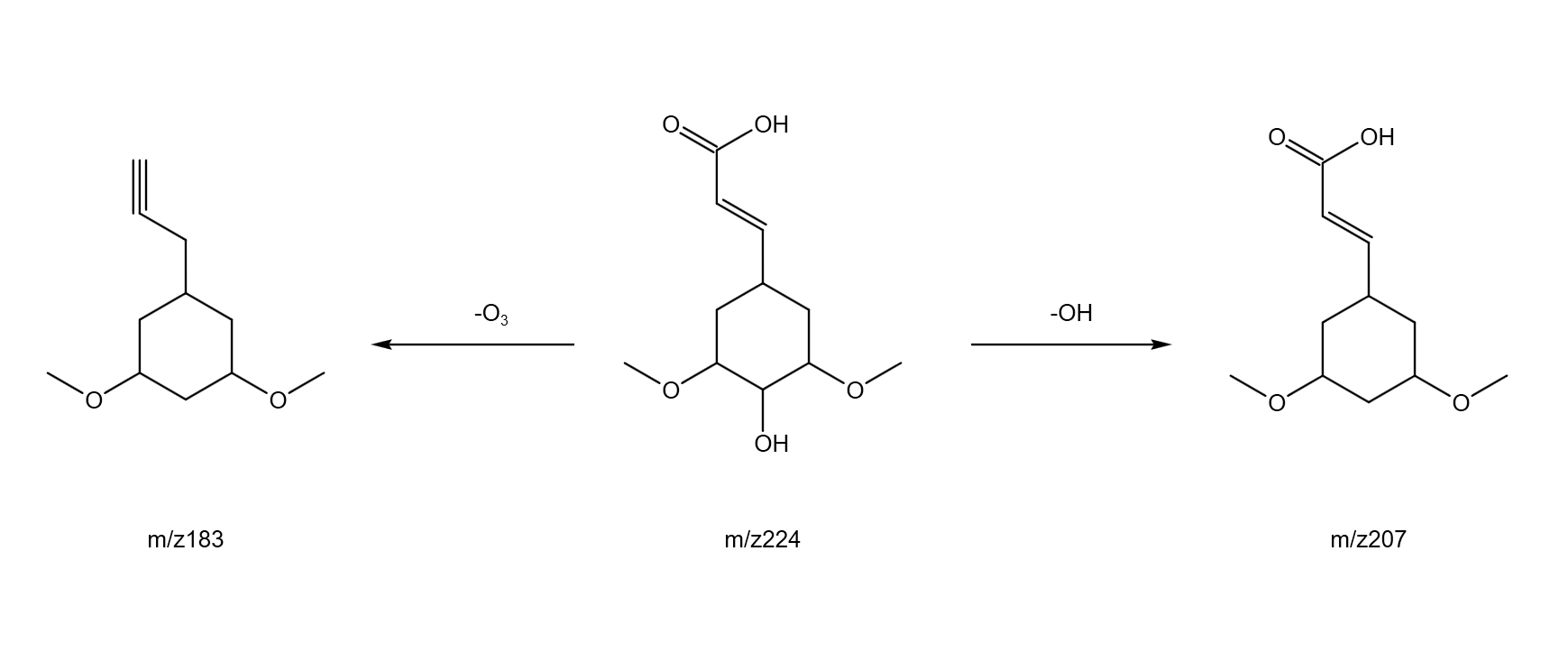


Supplementary Fig. 2 Spectrometry fragmentation modes of Sinapic Acid in positive ion mode.


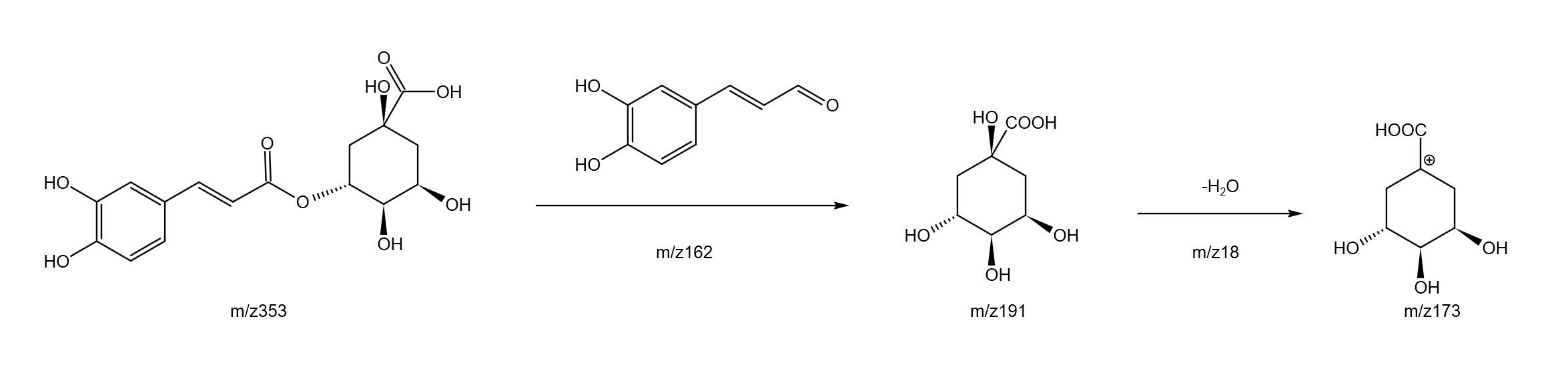


Supplementary Fig. 3 Spectrometry fragmentation modes of Chlorogenic Acid in positive ion mode.


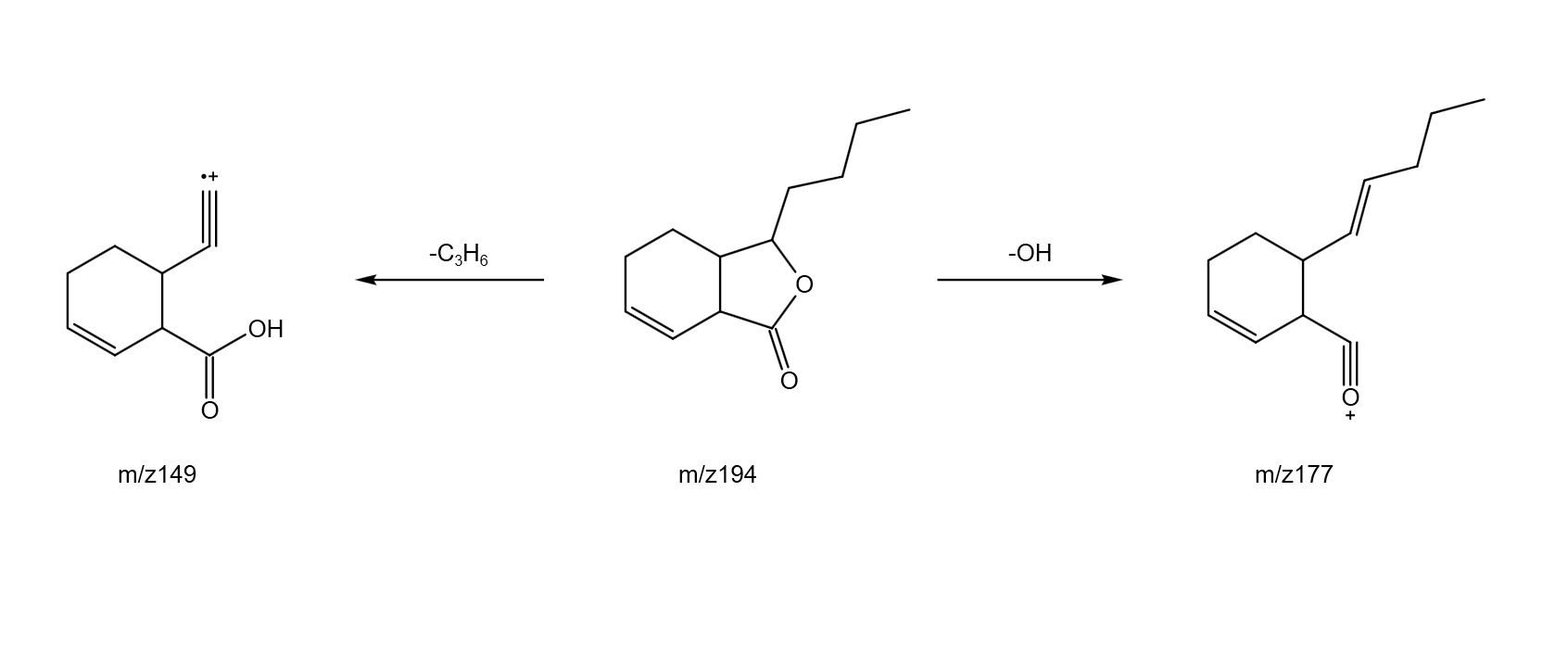


Supplementary Fig. 4 Spectrometry fragmentation modes of Cnidilide in positive ion mode.


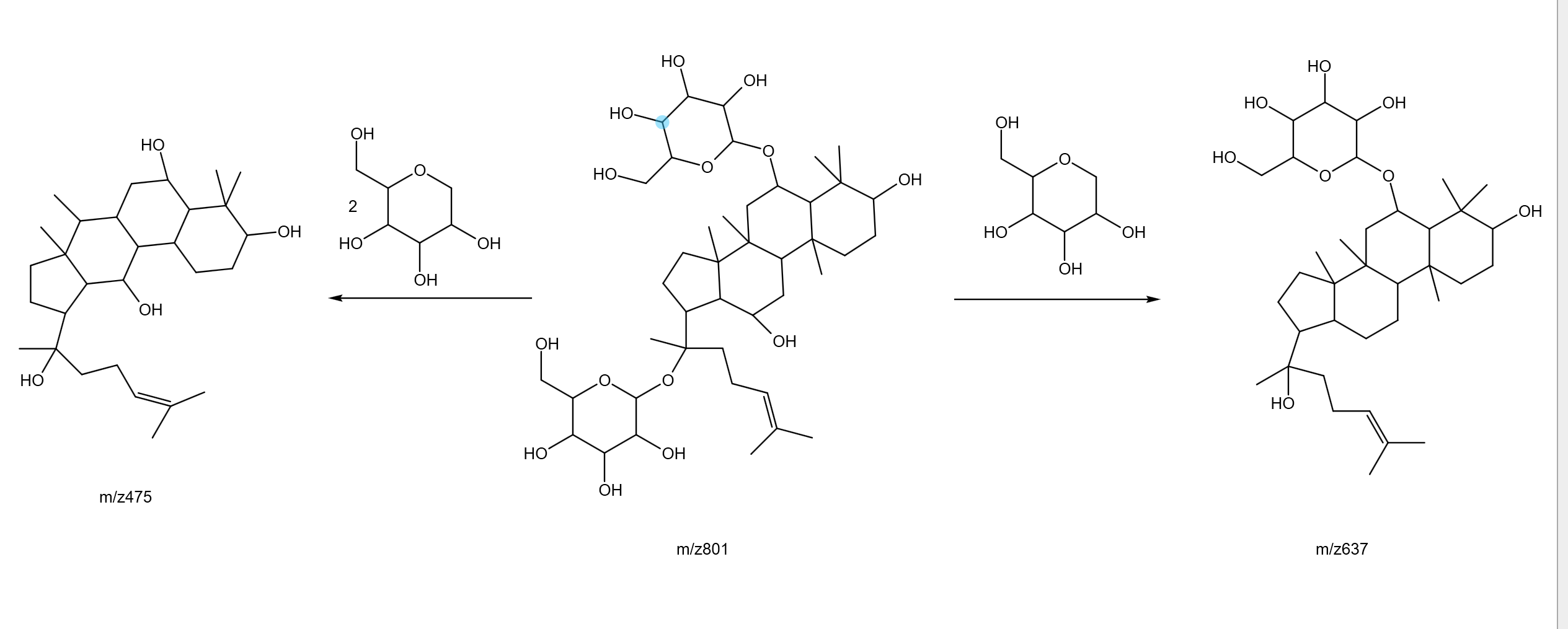


Supplementary Fig. 5 Spectrometry fragmentation modes of Ginsenoside Rg1 in positive ion mode.


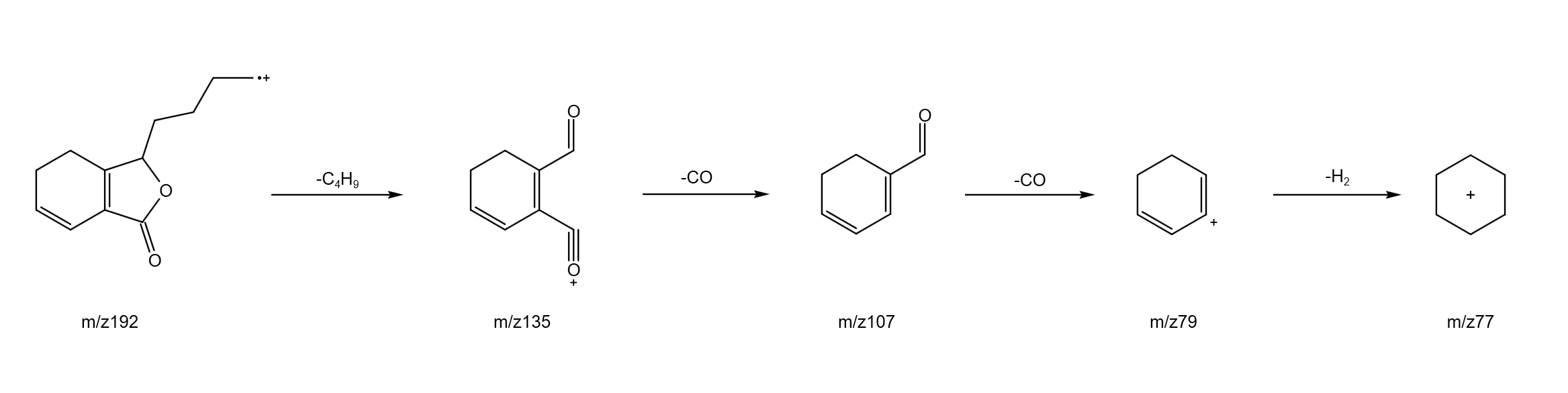


Supplementary Fig. 6 Spectrometry fragmentation modes of Senkyunolide A in positive ion mode.


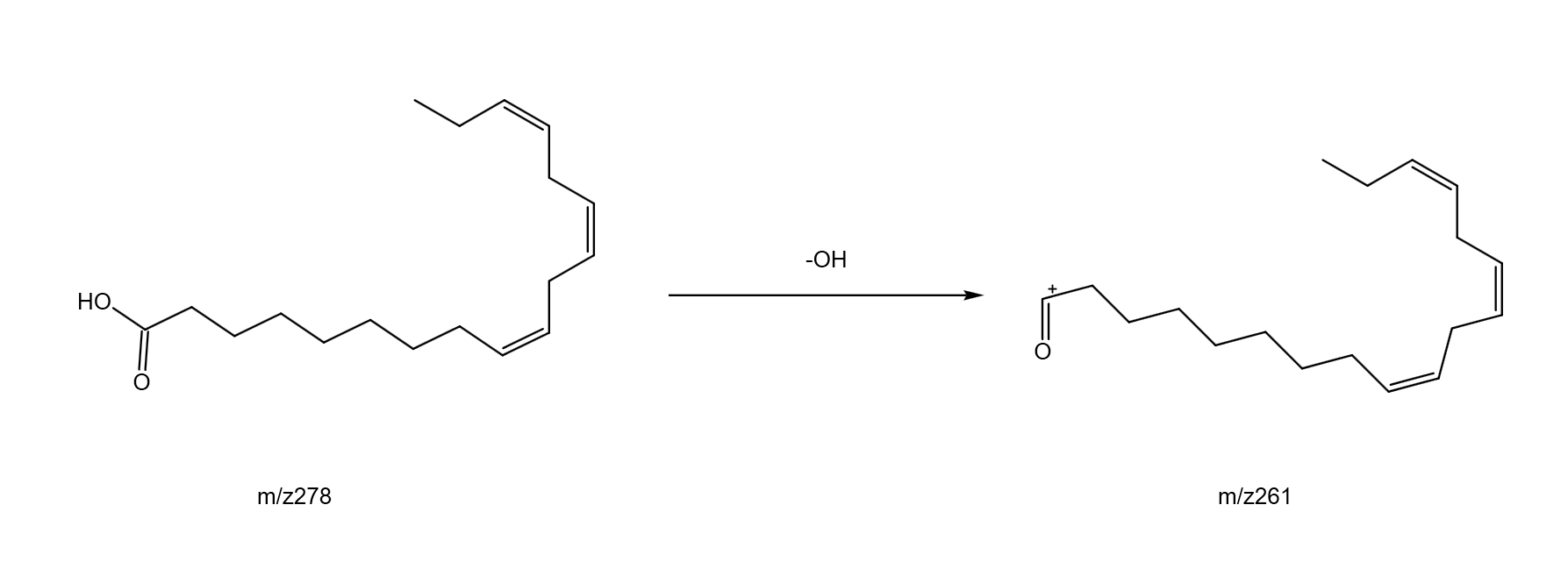


Supplementary Fig. 7 Spectrometry fragmentation modes of Linoleic Acid in positive ion mode.


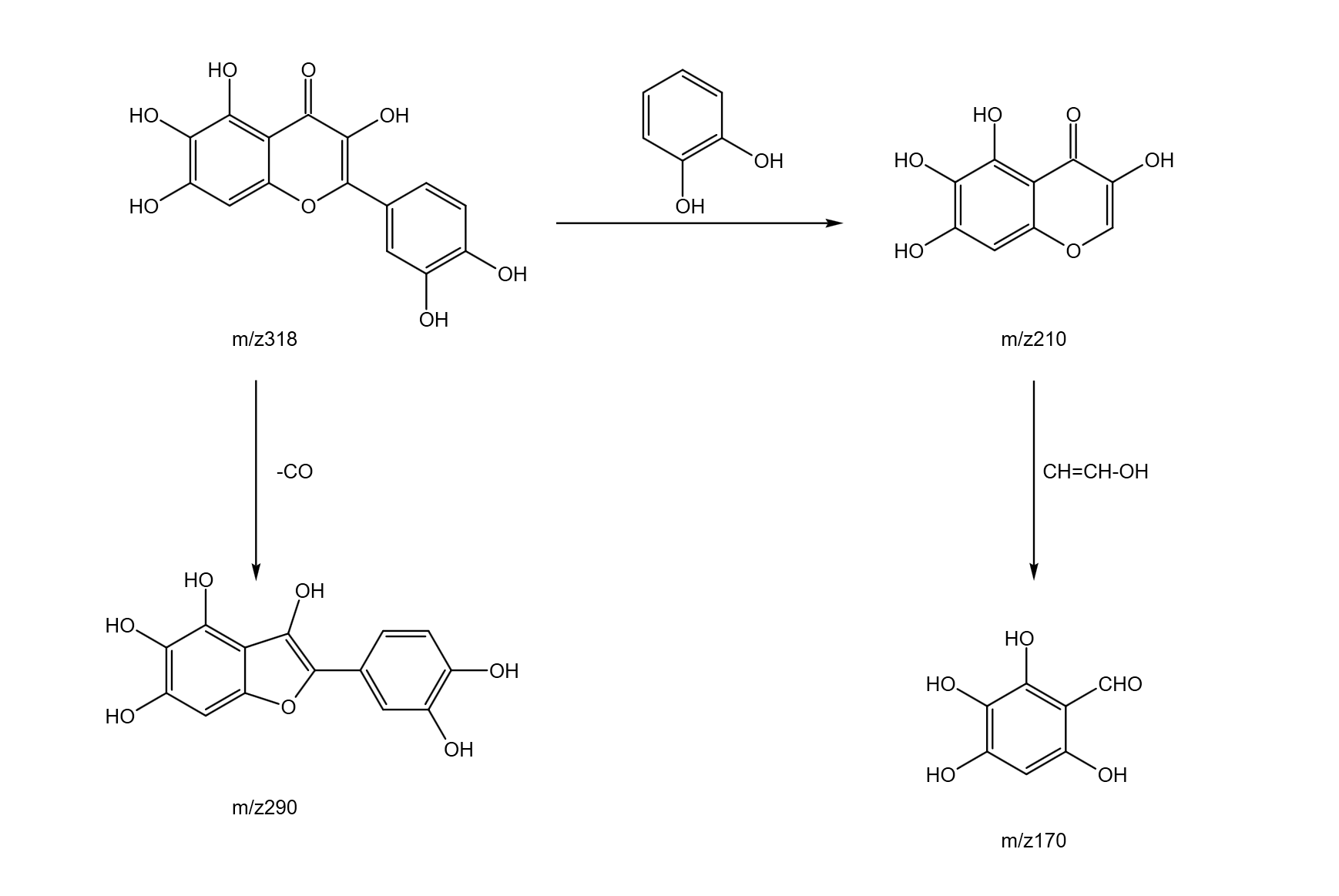


Supplementary Fig. 8 Spectrometry fragmentation modes of Quercetagetin in positive ion mode.


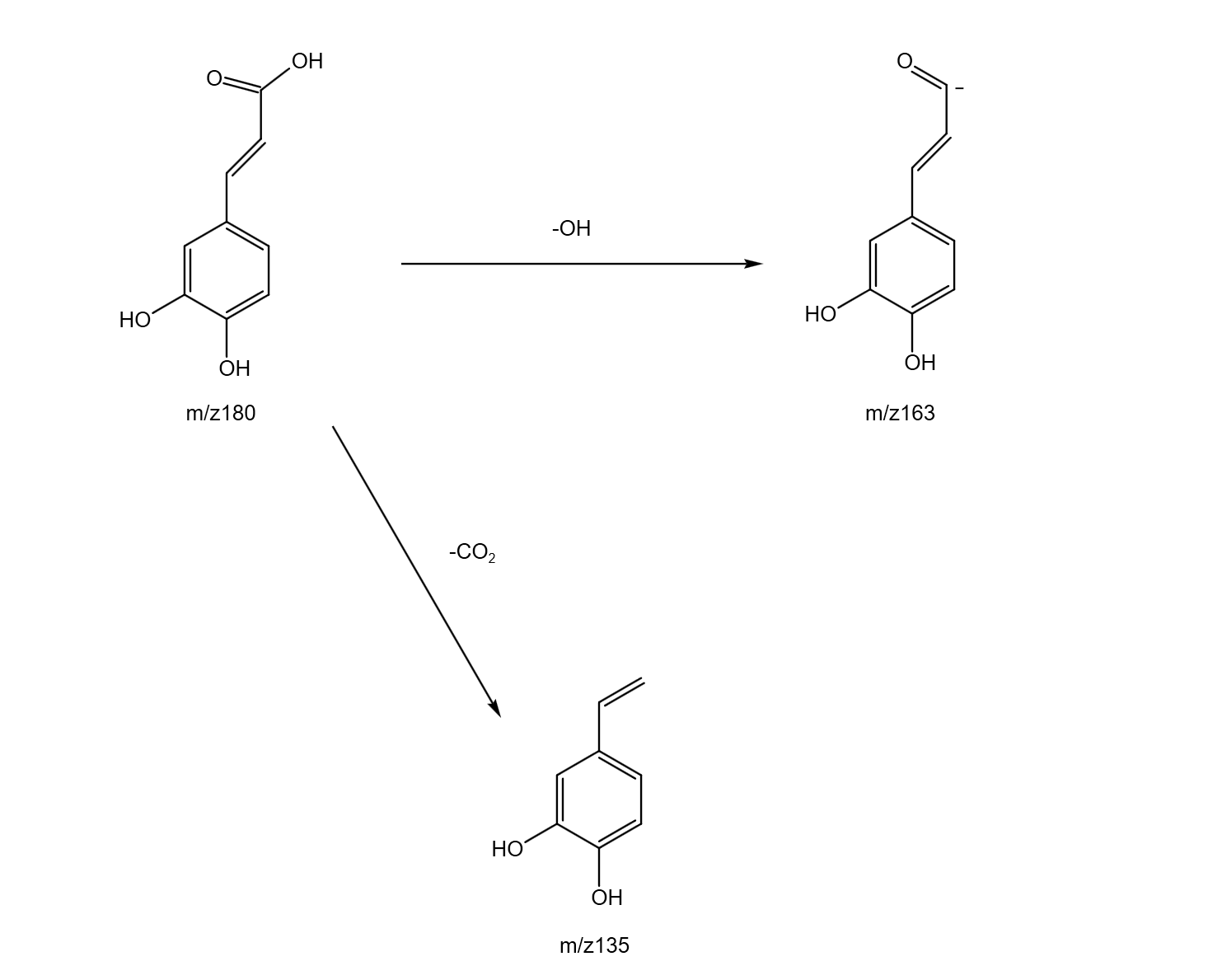


Supplementary Fig. 9 Spectrometry fragmentation modes of Caffeic Acid in positive ion mode.


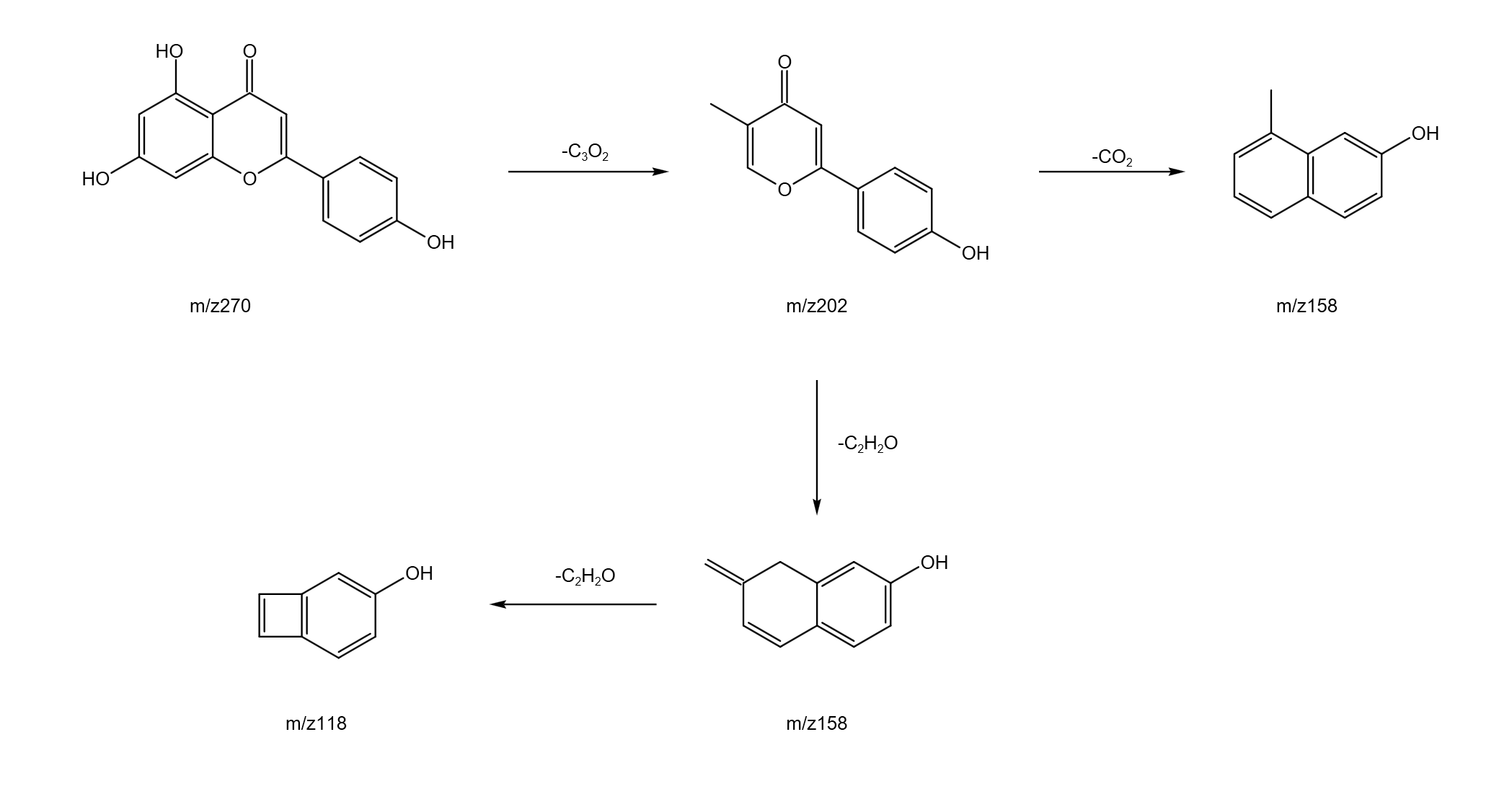


Supplementary Fig. 10 Spectrometry fragmentation modes of Apigenin in positive ion mode.


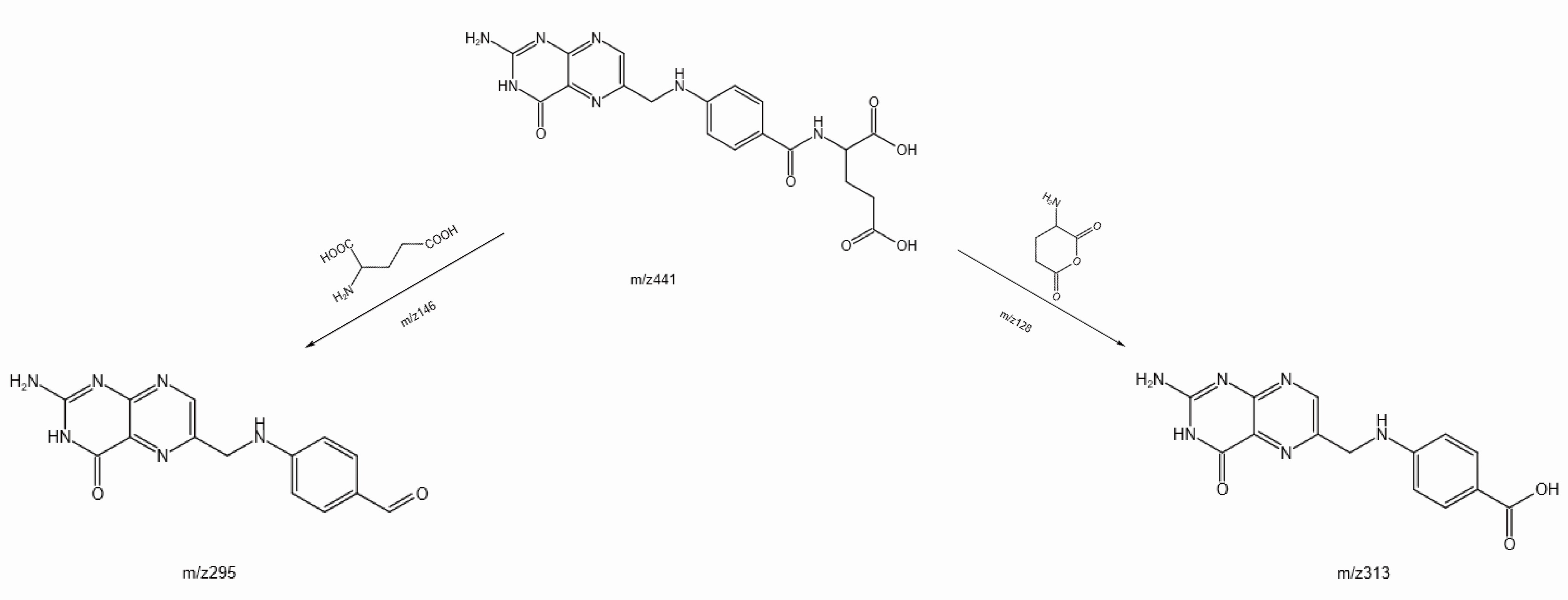


Supplementary Fig. 11 Spectrometry fragmentation modes of Folic Acid in positive ion mode.


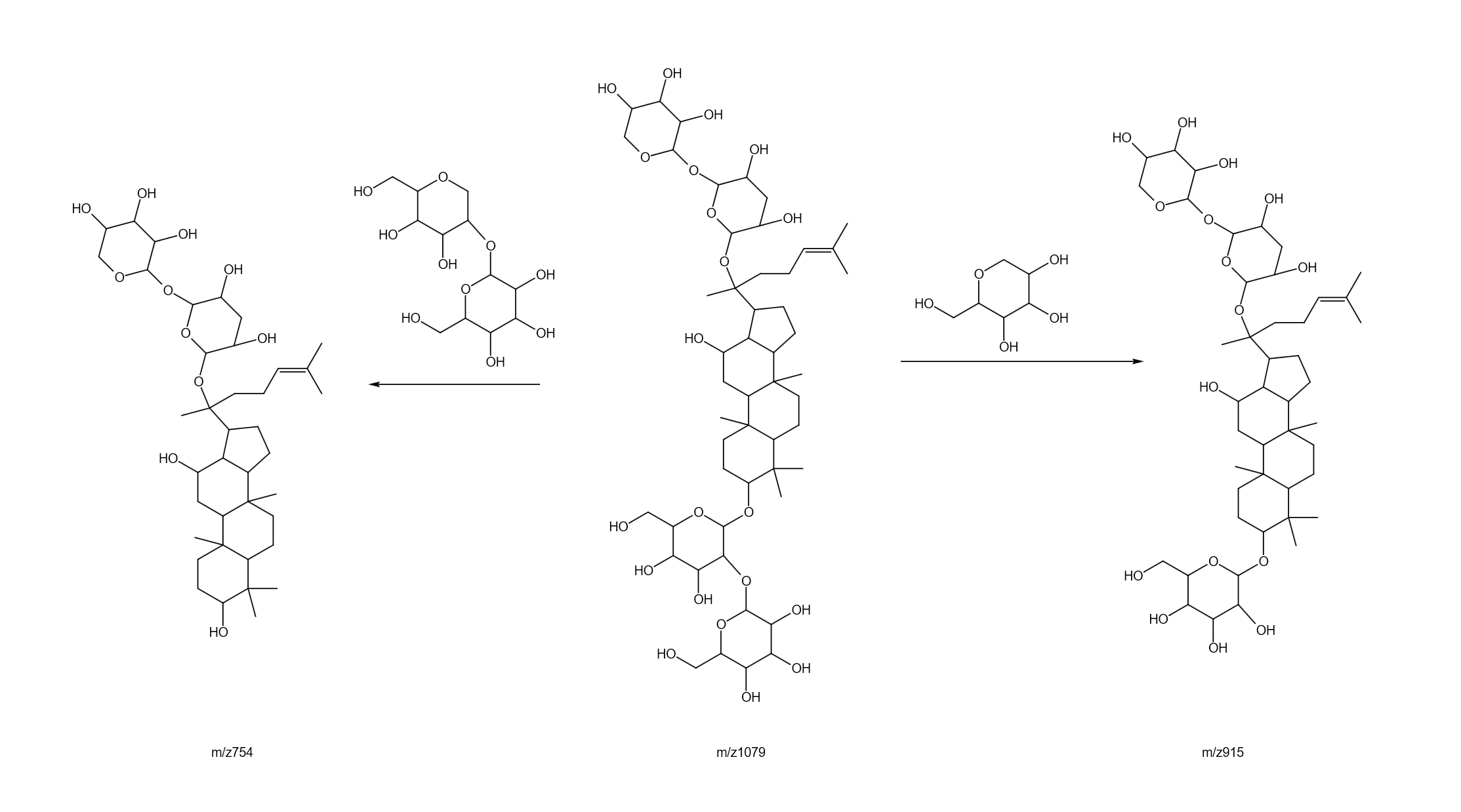


Supplementary Fig. 12 Spectrometry fragmentation modes of Ginsenoside Rb2 in positive ion mode.


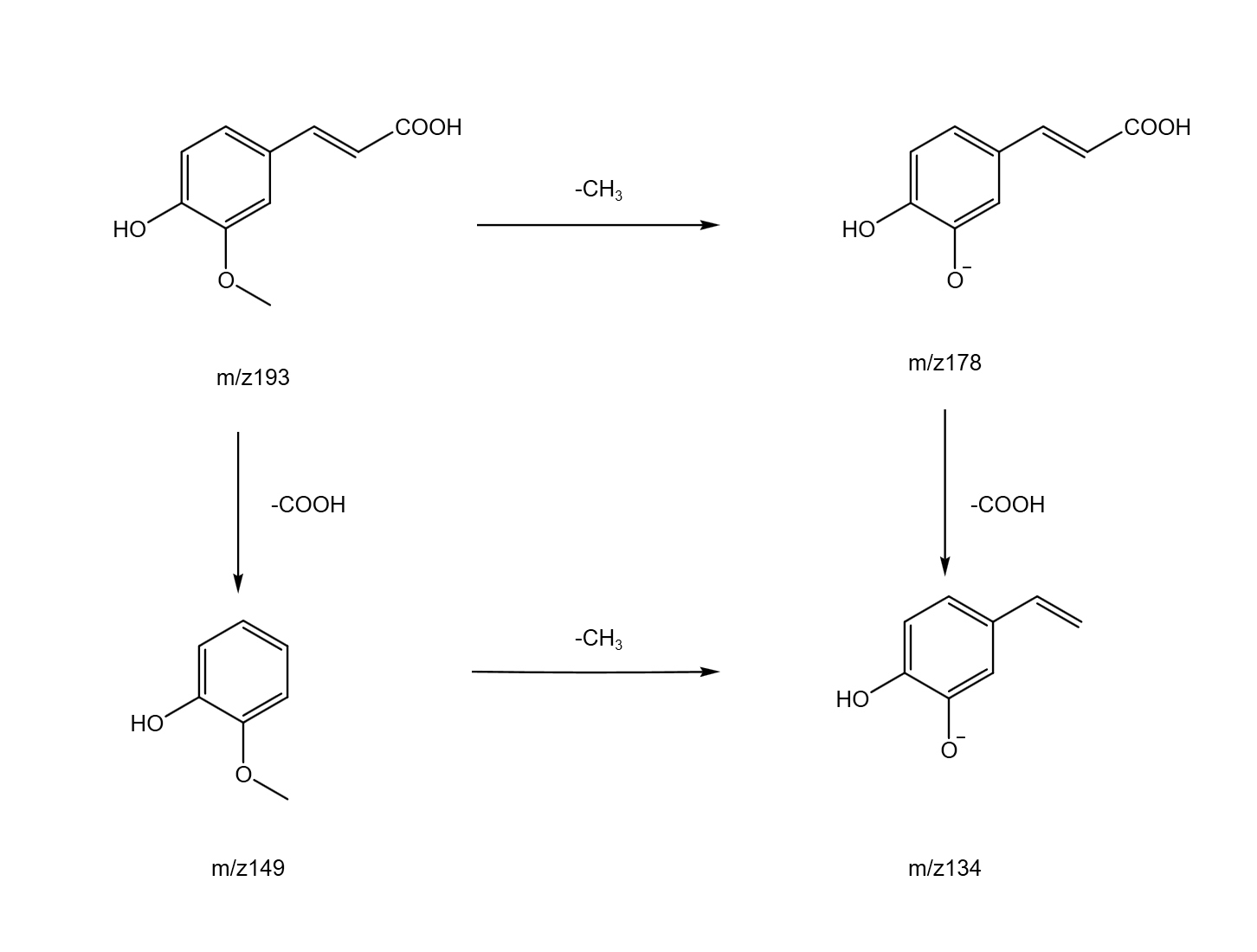


Supplementary Fig. 13 Spectrometry fragmentation modes of Ferulic Acid in positive ion mode.


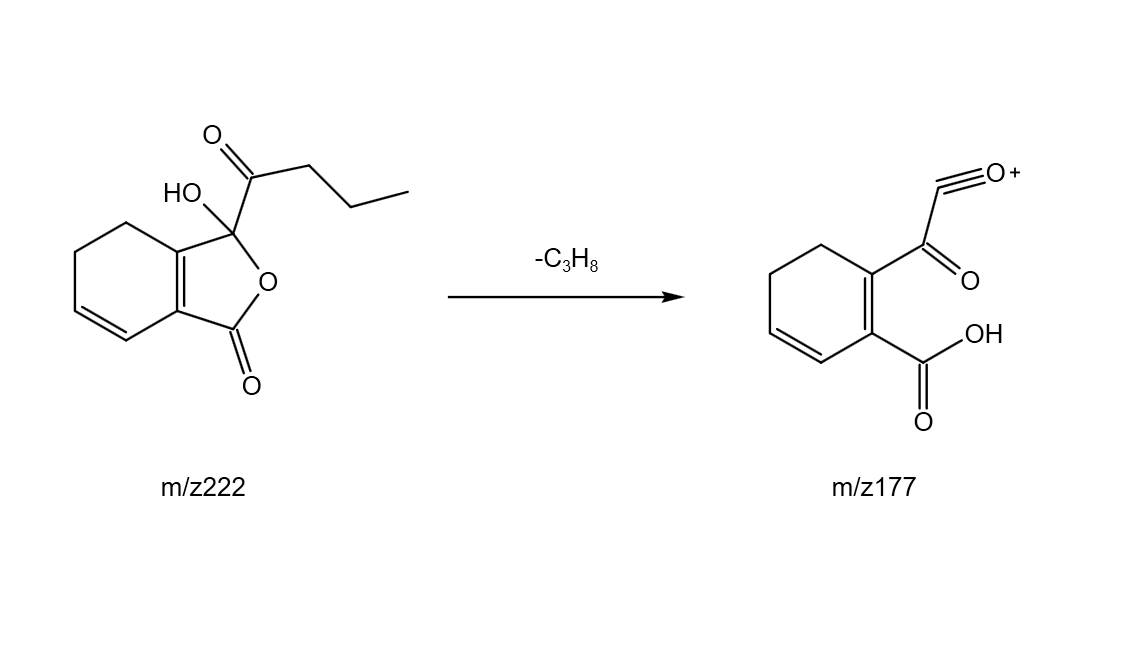


Supplementary Fig. 14 Spectrometry fragmentation modes of Senkyunolide D in positive ion mode.


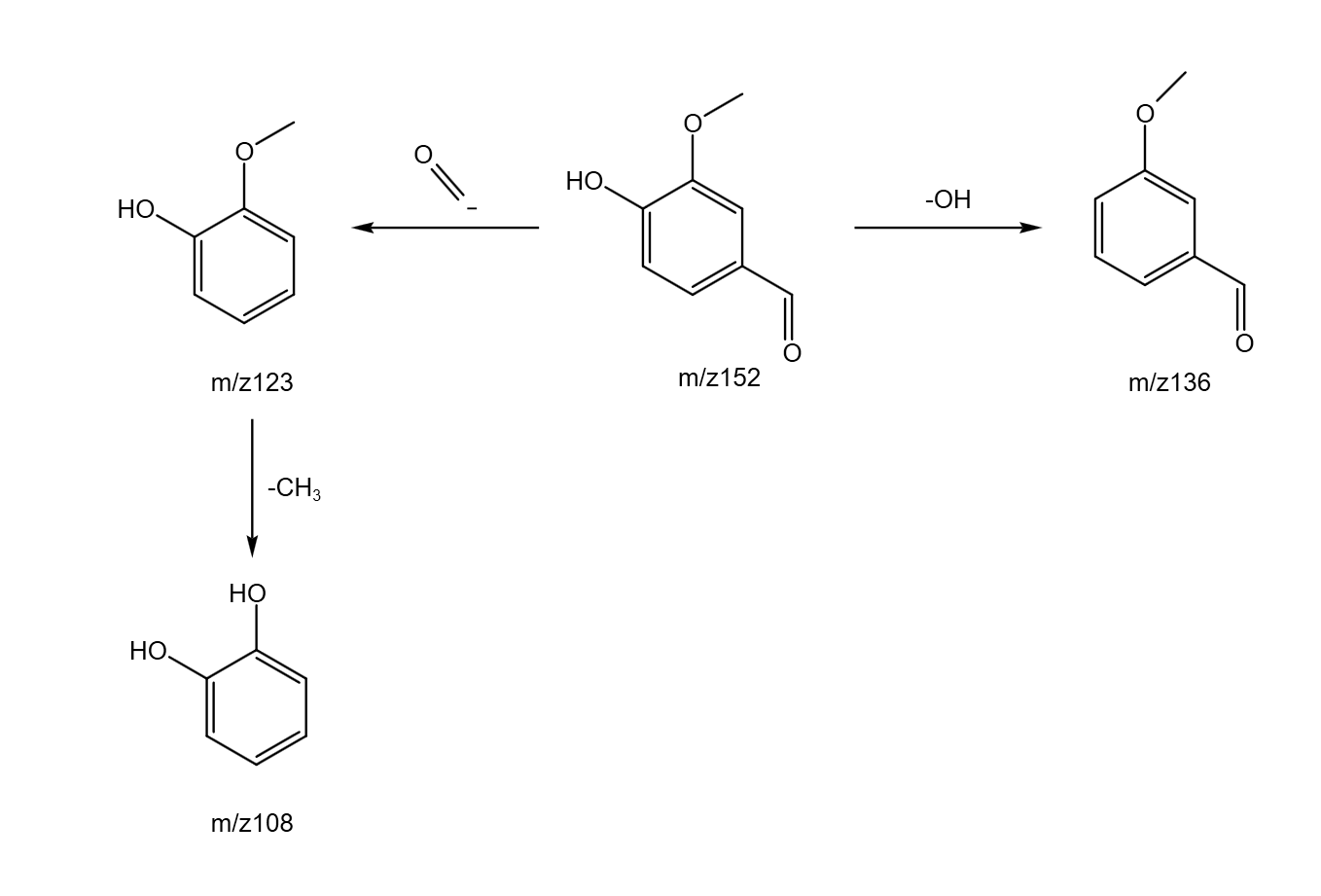


Supplementary Fig. 15 Spectrometry fragmentation modes of Vanillin in positive ion mode.


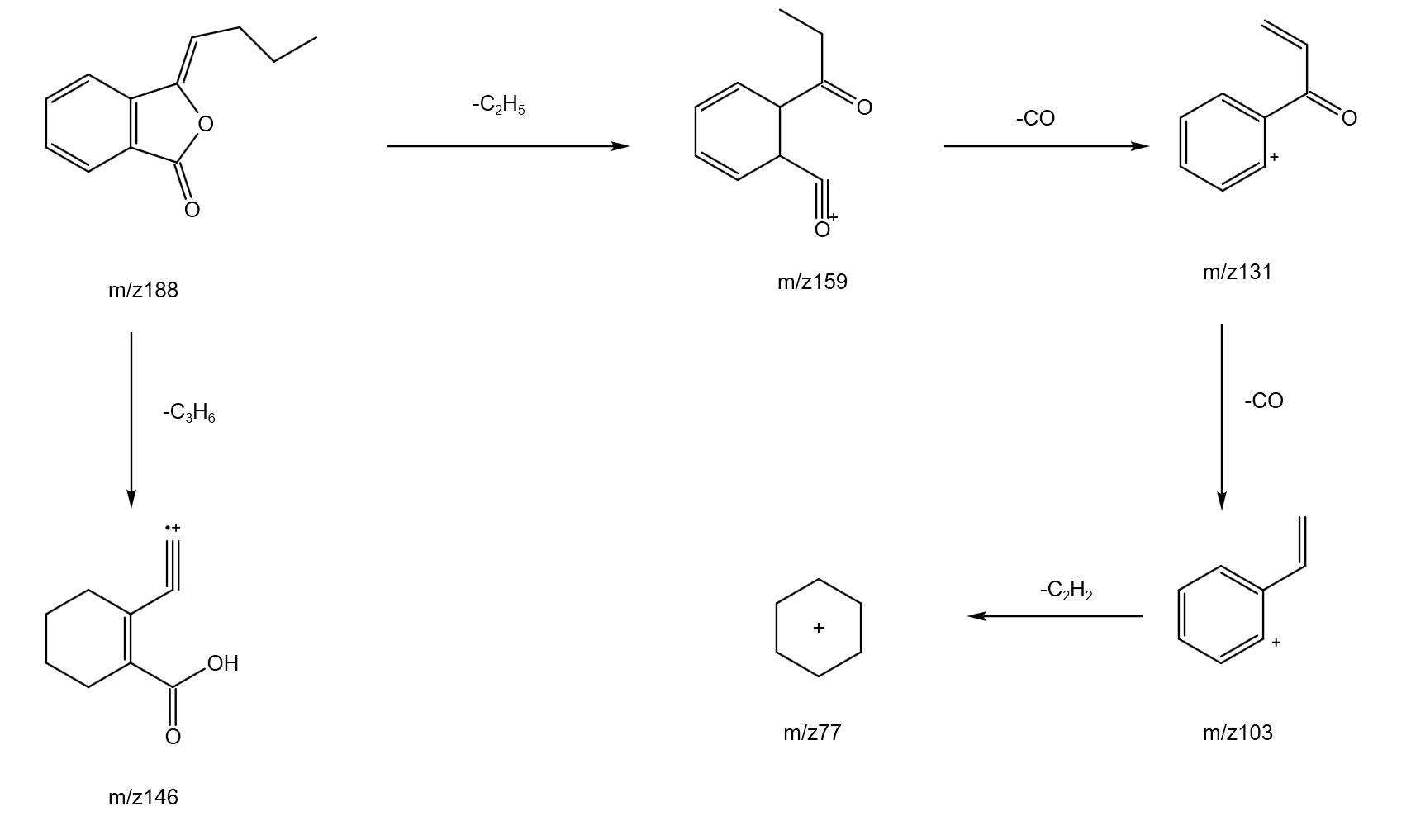


Supplementary Fig. 16 Spectrometry fragmentation modes of Butylidenephthalide in positive ion mode.


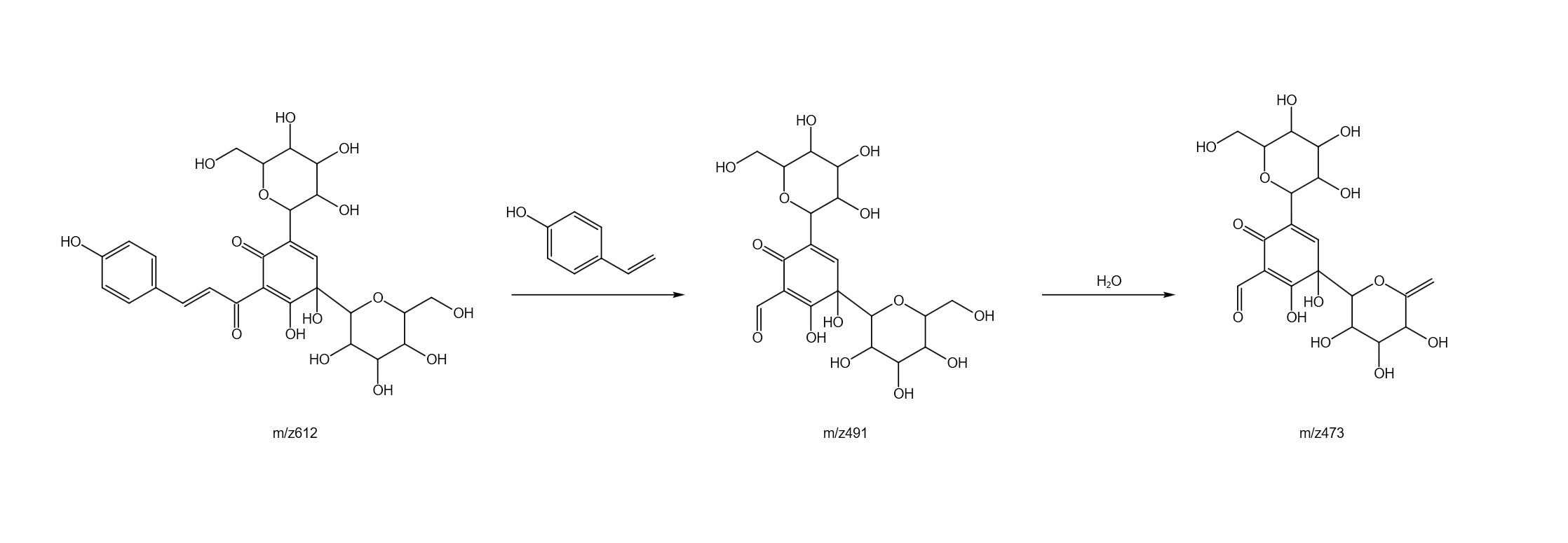


Supplementary Fig. 17 Spectrometry fragmentation modes of Hydroxysafflor Yellow A in positive ion mode.


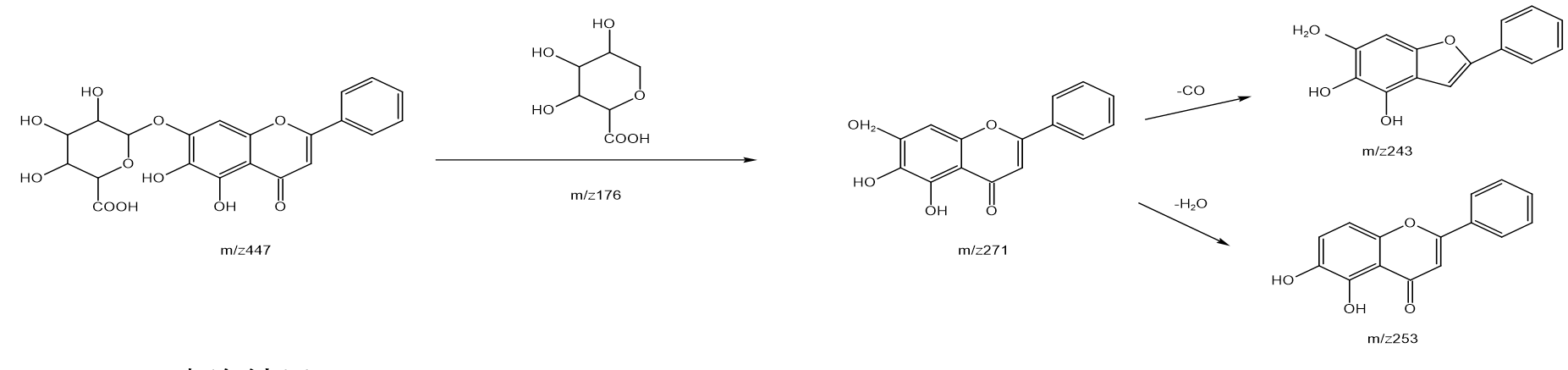


Supplementary Fig. 18 Spectrometry fragmentation modes of Baicalin in positive ion mode.


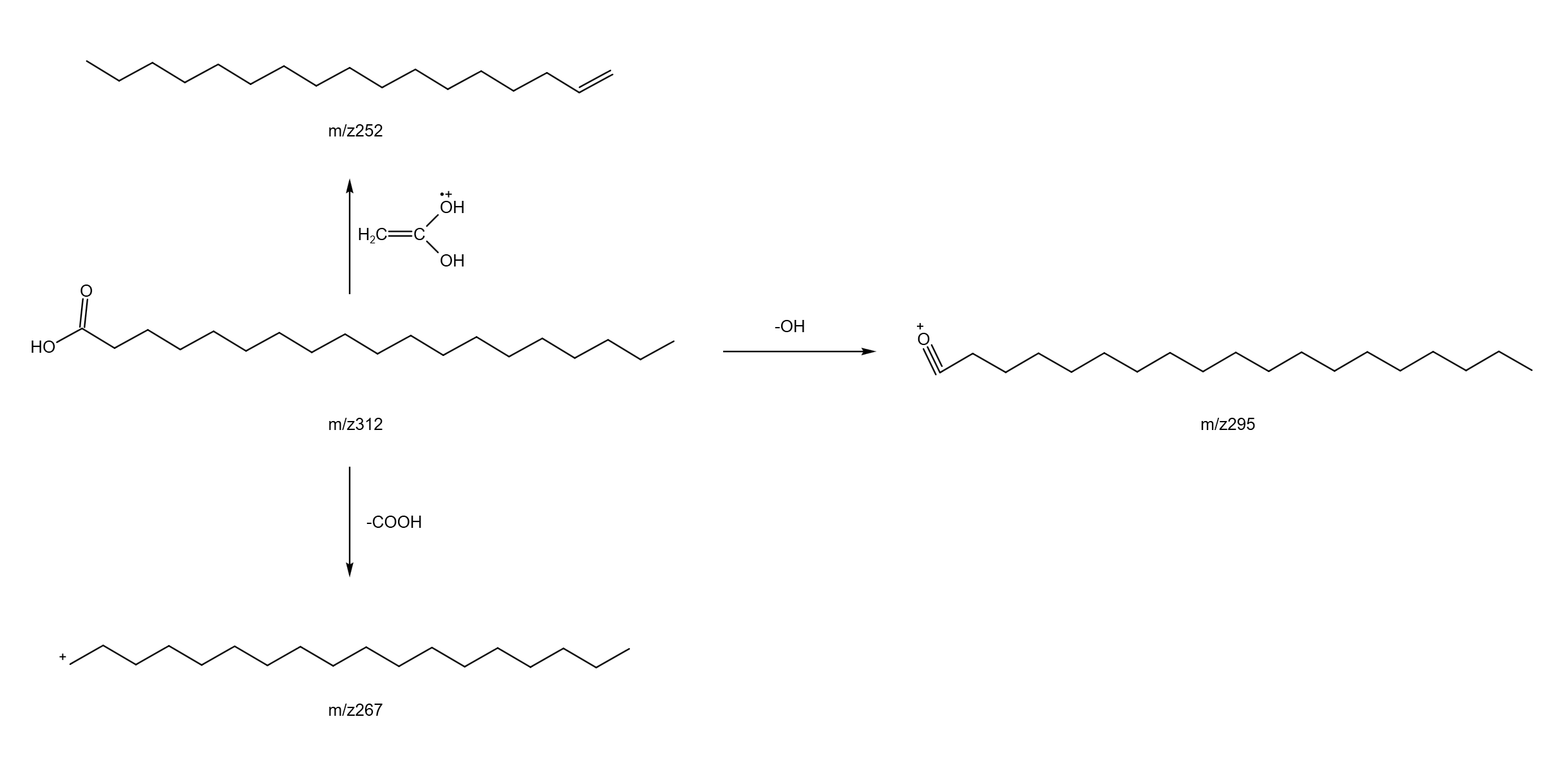


Supplementary Fig. 19 Spectrometry fragmentation modes of Arachic Acid in positive ion mode.


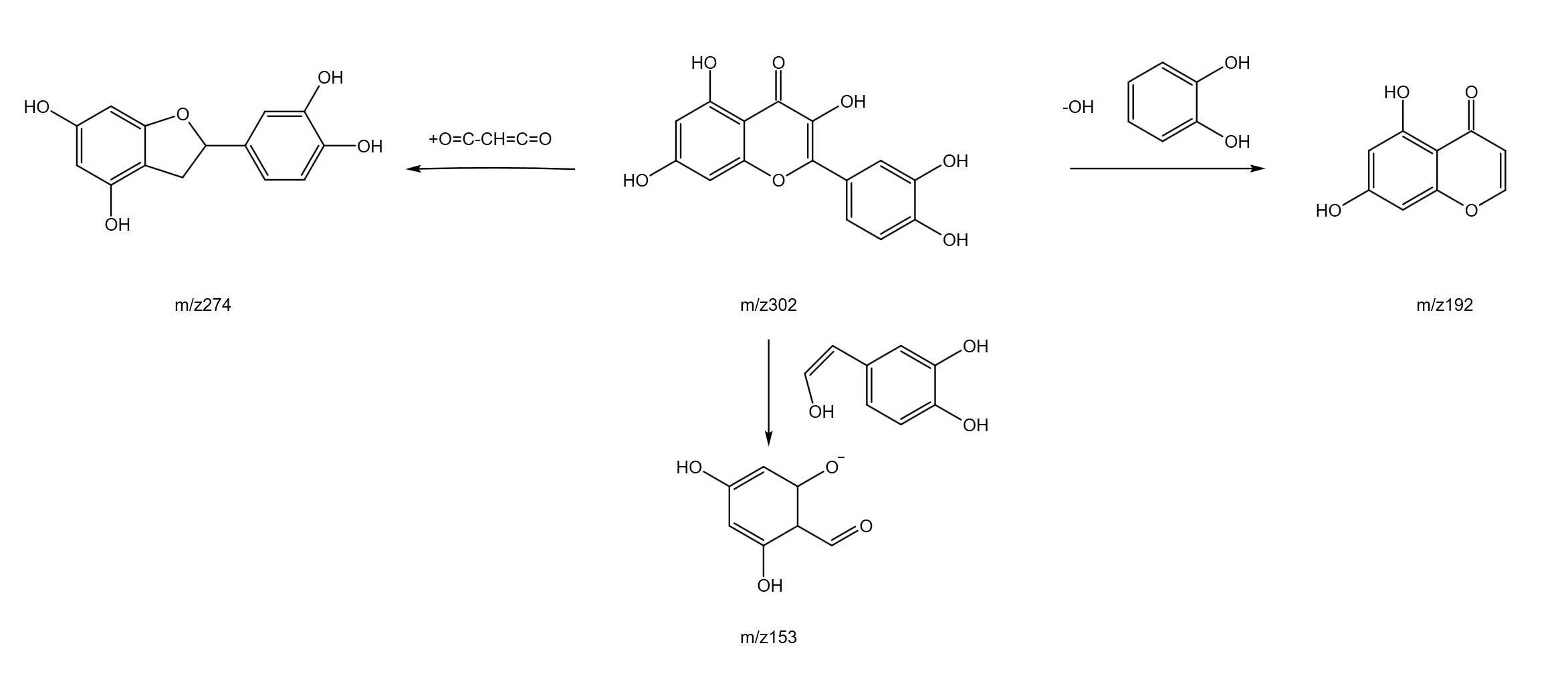


Supplementary Fig. 20 Spectrometry fragmentation modes of Quercetin in positive ion mode.


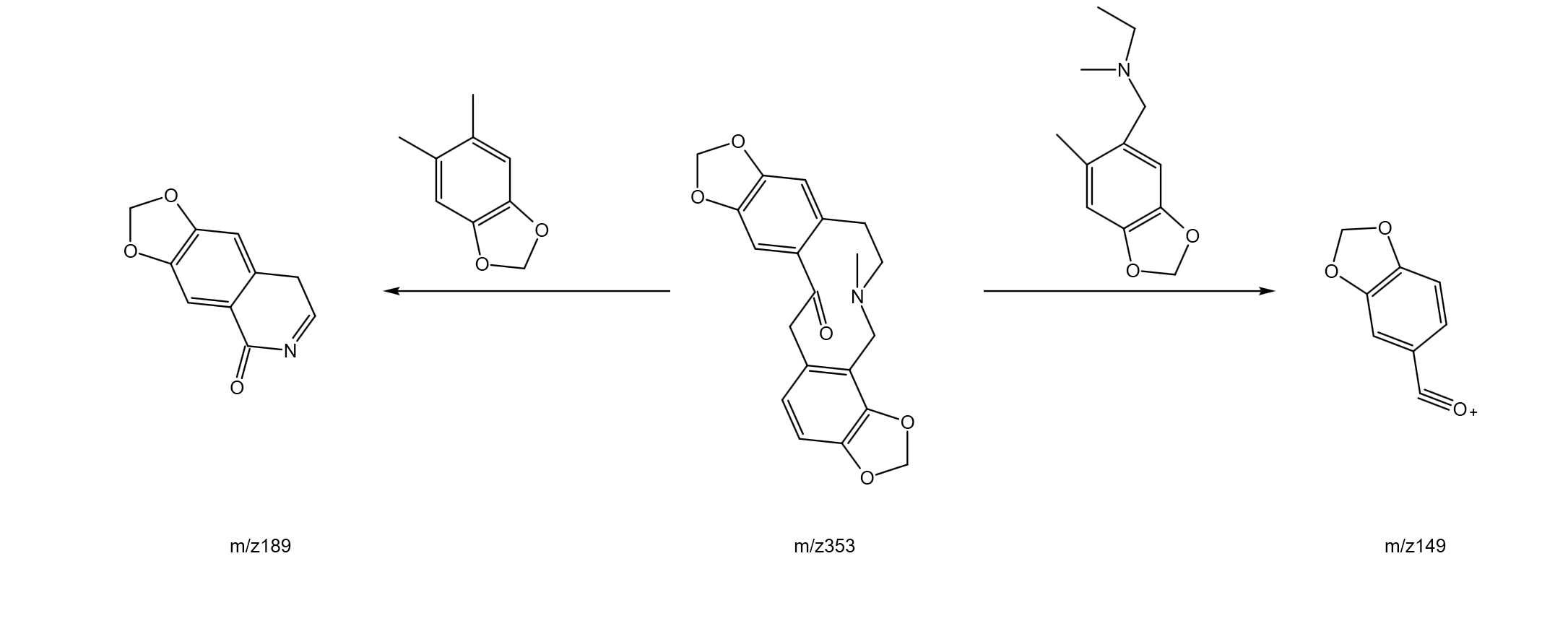


Supplementary Fig. 21 Spectrometry fragmentation modes of Fumarine in n positive ion mode.


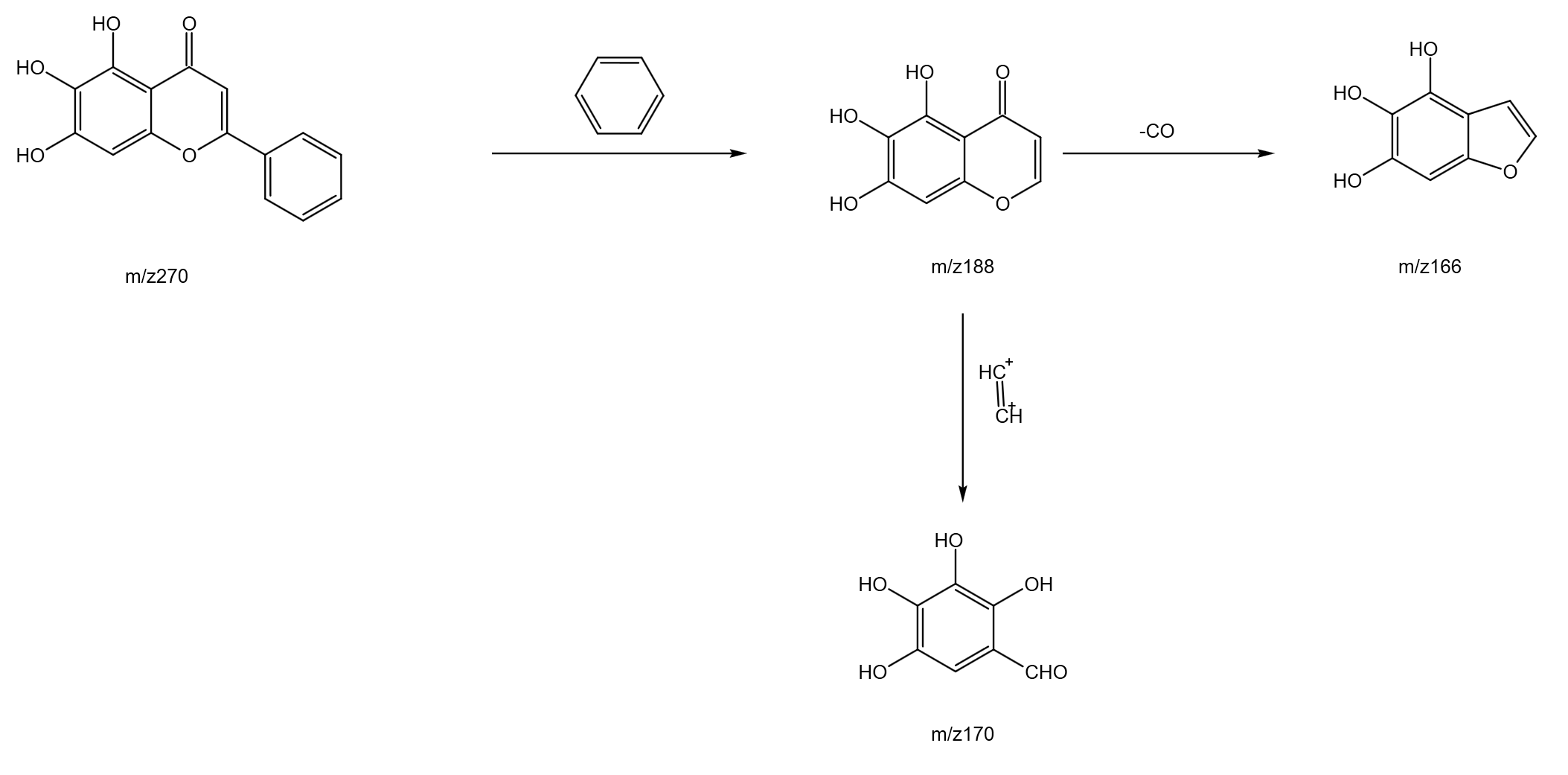


Supplementary Fig. 22 Spectrometry fragmentation modes of Baicalein in positive ion mode.


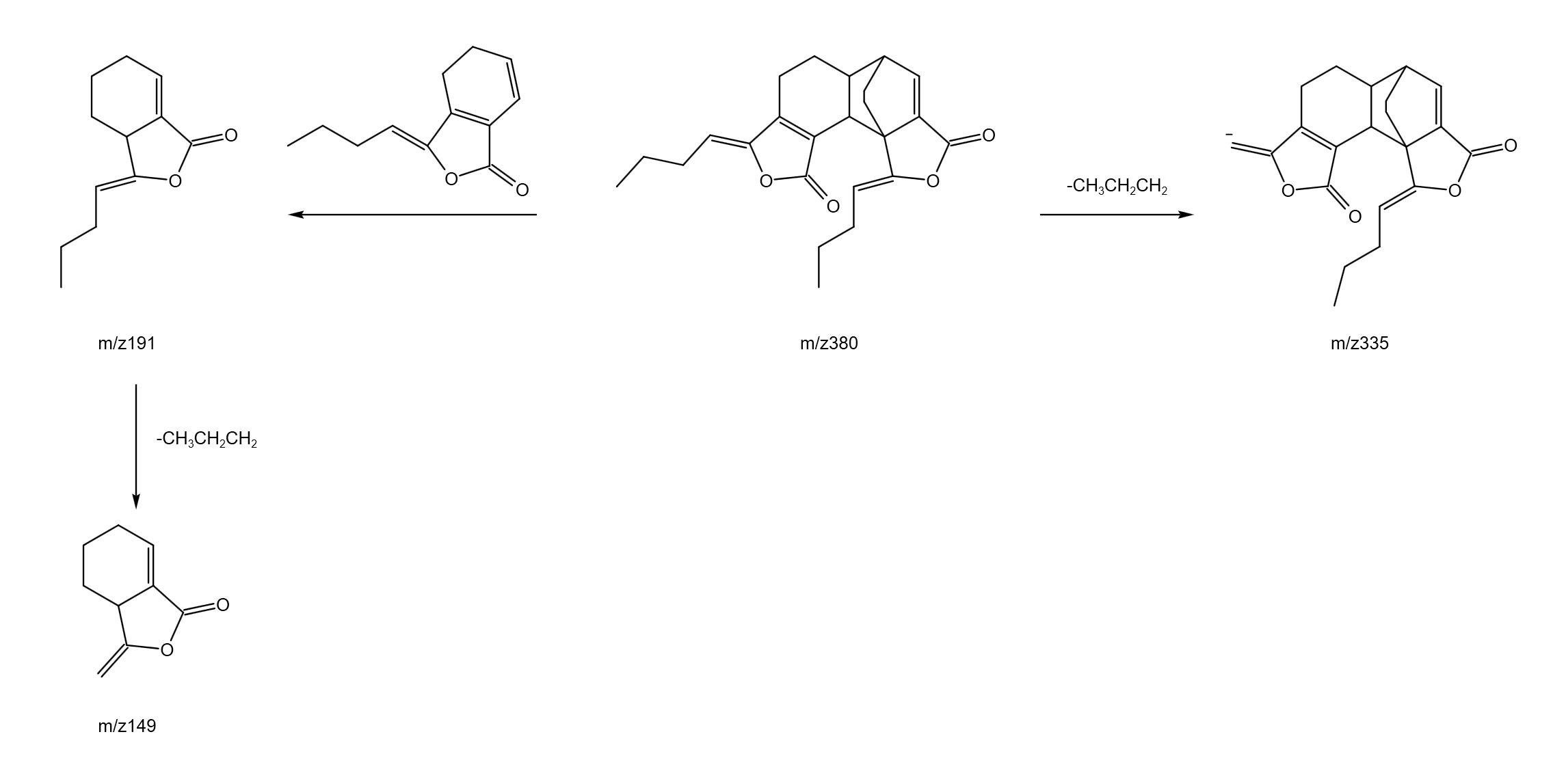


Supplementary Fig. 23 Spectrometry fragmentation modes of Levistolid A in positive ion mode.


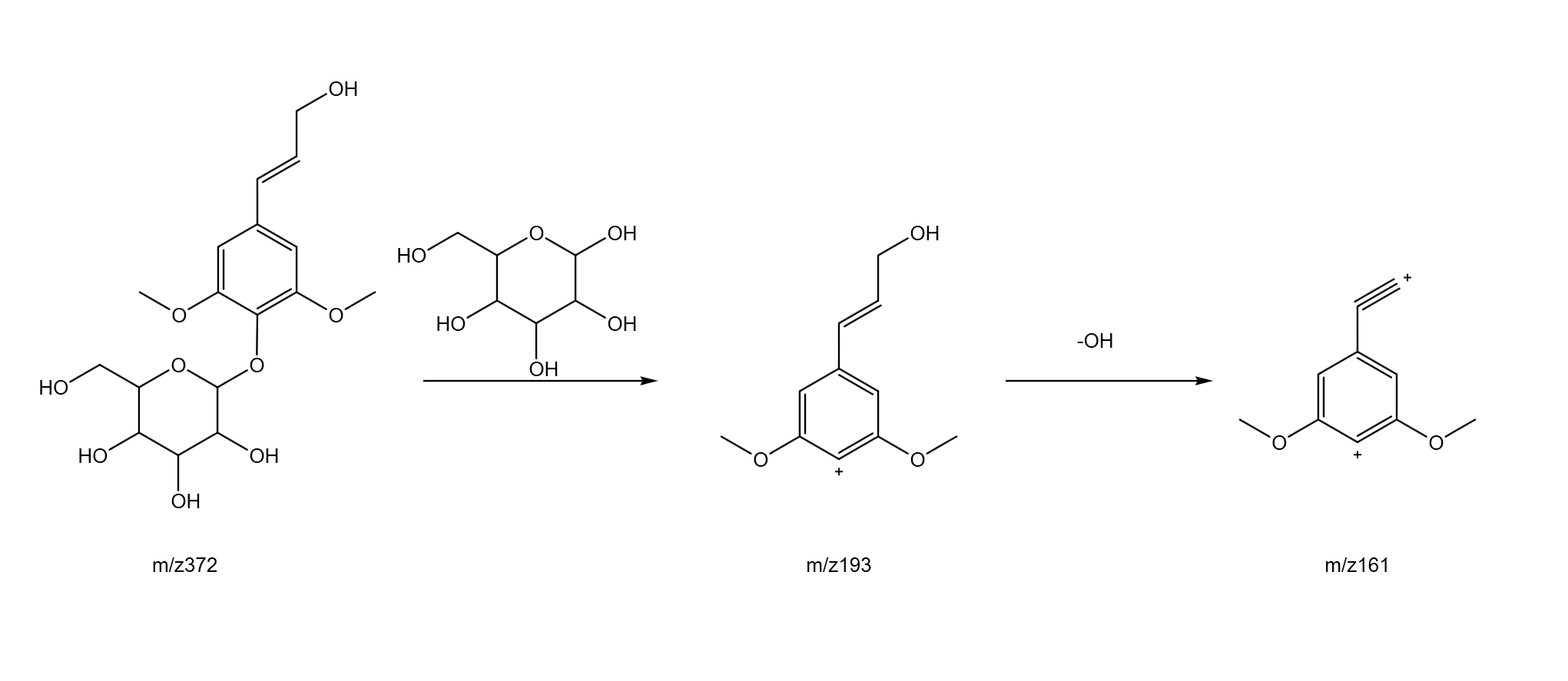


Supplementary Fig. 24 Spectrometry fragmentation modes of Syringin in positive ion mode.


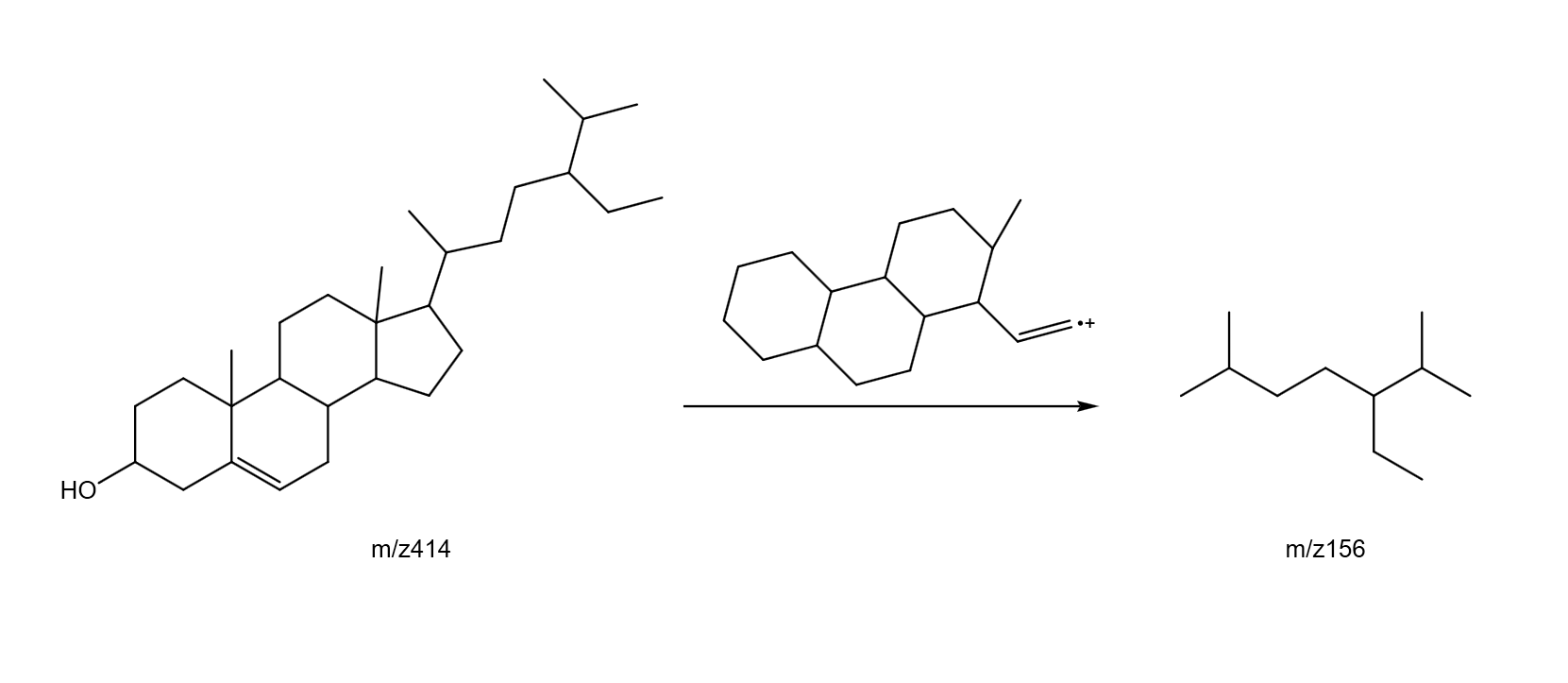


Supplementary Fig. 25 Spectrometry fragmentation modes of Beta-sitosterol in positive ion mode.


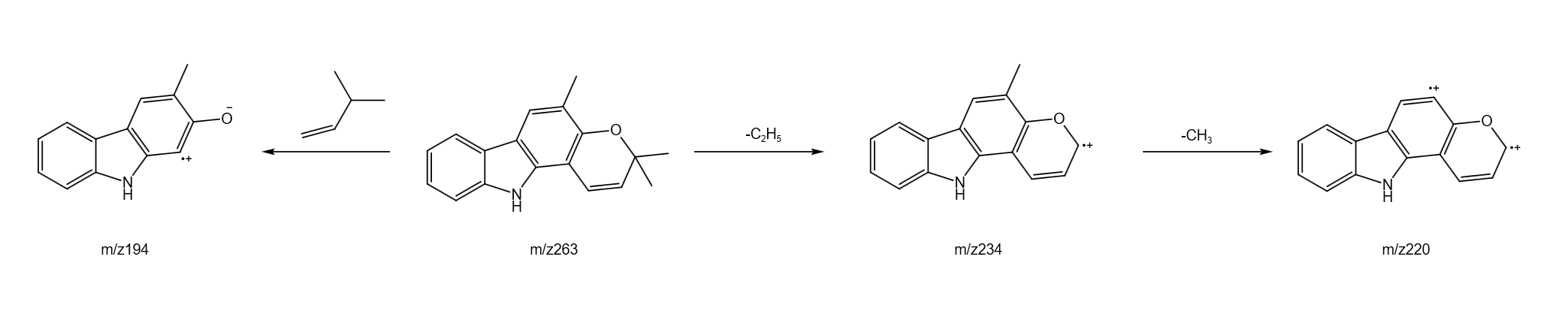


Supplementary Fig. 26 Spectrometry fragmentation modes of Girinimbine in positive ion mode.


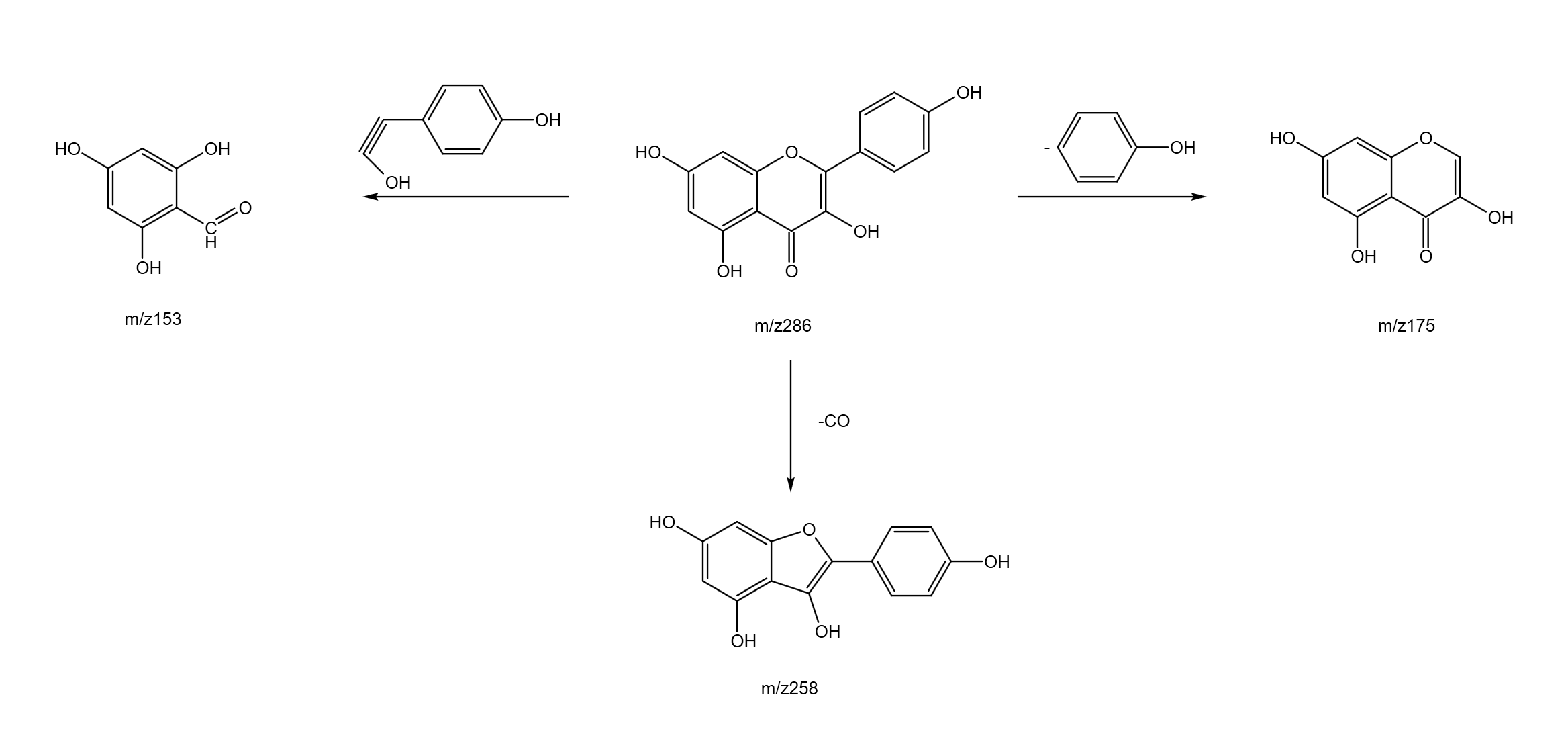


Supplementary Fig. 27 Spectrometry fragmentation modes of Kaempferol in positive ion mode.


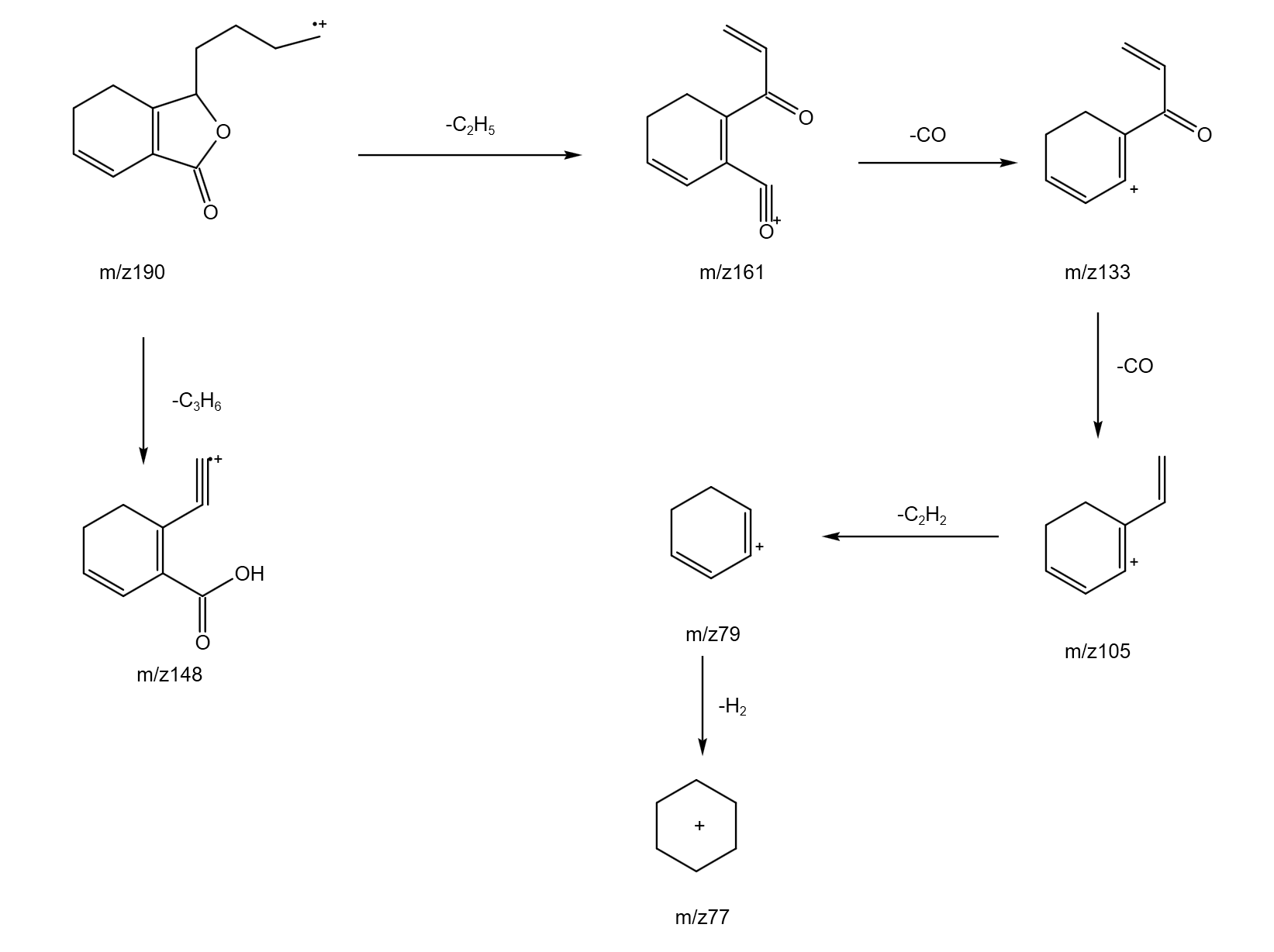


Supplementary Fig. 28 Spectrometry fragmentation modes of (*Z*)-Ligustilide in positive ion mode.
